# Driver Mutation Subtypes Differentially Shape Immune Evasion Landscapes in Melanoma: An AI‐Driven Inflammatory Pathway Model Implicating CCNE1

**DOI:** 10.1155/humu/6776070

**Published:** 2026-06-27

**Authors:** Chong Mao, Guobin Chen, Jiayu Tang, Rao Leng, Xiyuan Zhou, Jianing Yang

**Affiliations:** ^1^ Department of Dermatology, Sichuan Provincial People′s Hospital, University of Electronic Science and Technology of China, Chengdu, China, uestc.edu.cn; ^2^ Department of Rehabilitation, Sichuan Provincial People′s Hospital, University of Electronic Science and Technology of China, Chengdu, China, uestc.edu.cn; ^3^ School of Medicine, University of Electronic Science and Technology of China, Chengdu, China, uestc.edu.cn

**Keywords:** CCNE1, machine learning, melanoma, prognostic model, tumor immune microenvironment

## Abstract

**Background:**

Melanoma harbors highly heterogeneous tumor immune microenvironments shaped by driver mutations in BRAF, NRAS, and NF1. How mutational context modulates inflammatory signaling and immune evasion mechanisms of prognosis‐related genes remains poorly understood.

**Methods:**

ssGSEA scored 15 inflammatory pathways across four cohorts (TCGA, GSE19234, GSE22153, and GSE65904). Cross‐cohort univariate Cox regression and a 10‐algorithm machine learning framework identified and optimized a prognostic model. Immune microenvironment differences across BRAF, NRAS, NF1, and Triple‐WT subtypes were characterized using ESTIMATE, CIBERSORT, and GSVA. CCNE1 was validated by shRNA knockdown in melanoma cell lines, and a mutation subtype–specific virtual knockdown model was constructed from scRNA‐seq data.

**Results:**

The prognostic model achieved a 5‐year AUC of 0.95 in TCGA and outperformed published signatures in three of four cohorts. High‐risk patients showed markedly reduced immune infiltration (ImmuneScore *r* = −0.51) and an immunosuppressive phenotype. Mutation subtype analysis revealed distinct immune landscapes: NF1‐mutant tumors showed the highest antigen presentation and IFN‐*γ* pathway enrichment; BRAF‐mutant tumors displayed the highest stromal score and M0 macrophage proportions; and NRAS‐mutant tumors exhibited the lowest NK cell activity and most pronounced immunosuppression. CCNE1 was the gene most strongly correlated with the risk score and was validated as an independent poor prognostic marker across all cohorts. shRNA‐mediated knockdown inhibited migration and enhanced adhesion in melanoma cell lines. Virtual knockdown modeling showed that CCNE1 suppression upregulated antigen presentation genes (HLA‐A/B/C, B2M, and TAP1/2) and downregulated immune checkpoint molecules in a mutation subtype–dependent manner, with the strongest proimmunogenic effects in NF1‐mutant cells and LAG3 downregulation predominantly in BRAF‐mutant cells.

**Conclusion:**

This study establishes a high‐performance inflammatory pathway–based prognostic model for melanoma and demonstrates that driver mutation subtypes differentially shape the immune microenvironment landscape. CCNE1 functions as a key oncogenic immune regulator whose modulation of antigen presentation and immune checkpoint expression is contingent on mutational context, most prominently in NF1‐mutant tumors, underscoring the value of mutation‐informed personalized immunotherapy strategies in melanoma.

## 1. Introduction

Melanoma is one of the most aggressive and lethal malignancies arising from the malignant transformation of melanocytes, accounting for the vast majority of skin cancer–related mortality despite representing only a small fraction of all skin cancer diagnoses [1]. Globally, the incidence of melanoma has risen steadily over recent decades, posing a significant public health burden [2]. Although the advent of immune checkpoint inhibitors (ICIs) targeting PD‐1/PD‐L1 and CTLA‐4, as well as BRAF/MEK‐targeted therapies, has substantially improved outcomes for a subset of patients, the prognosis of advanced and metastatic melanoma remains dismal, with a considerable proportion of patients exhibiting primary or acquired resistance to current therapeutic strategies [3, 4]. The biological complexity of melanoma, characterized by high tumor mutational burden (TMB), pronounced immune evasion, and extensive intratumoral heterogeneity, highlights the critical need for robust prognostic biomarkers capable of stratifying patients and guiding individualized clinical decision‐making [5]. Inflammatory signaling pathways—including TNF signaling, interferon (IFN) signaling, IL‐12/IL‐23 signaling, and JAK‐STAT signaling—constitute central regulatory axes of the tumor immune microenvironment (TME), governing immune cell recruitment, antigen presentation, cytokine production, and the balance between immune activation and suppression [6]. Dysregulation of these pathways has been broadly implicated in shaping the immunosuppressive TME and driving immune evasion across multiple malignancies [7]. Nevertheless, a comprehensive and systematic characterization of the inflammatory pathway landscape and its prognostic implications in melanoma, particularly across large and independent patient cohorts, has not been established.

The integration of high‐throughput transcriptomic profiling with advanced computational methodologies has transformed the landscape of cancer prognostic biomarker discovery [8–10]. Single‐sample gene set enrichment analysis (ssGSEA) enables quantification of pathway activity at the individual patient level, facilitating the dissection of complex inflammatory signaling landscapes across heterogeneous tumor samples [11]. Building upon such analytical frameworks, machine learning algorithms have emerged as powerful tools for constructing robust prognostic models in oncology, offering the capacity to integrate multidimensional molecular features and capture nonlinear associations with clinical outcomes [12]. In particular, ensemble frameworks integrating multiple survival prediction algorithms—such as Lasso‐Cox, Random Survival Forest (RSF), CoxBoost, and Gradient Boosting Machine (GBM)—have demonstrated substantially superior predictive performance and generalizability compared with conventional single‐algorithm approaches [13]. Despite these methodological advances, a systematic machine learning–based prognostic framework derived from inflammatory pathway gene signatures and rigorously validated across multiple independent melanoma cohorts remains lacking. Furthermore, TMB has emerged as a clinically relevant predictor of immunotherapy response in melanoma [14]; however, its combinatorial interaction with transcriptomic risk signatures in refining prognostic stratification has not been fully explored.

Cyclin E1 (CCNE1), a critical regulator of the G1/S cell cycle checkpoint, has been reported to be frequently amplified or overexpressed across multiple cancer types, where it promotes tumor cell proliferation, replication stress, and genomic instability [15, 16]. Beyond its canonical cell cycle function, accumulating evidence suggests that CCNE1 may exert broader oncogenic roles in regulating tumor cell migration, invasion, and remodeling of the tumor microenvironment [17]. Nevertheless, the mechanistic contribution of CCNE1 to melanoma progression, its association with immune microenvironment composition, and its functional role in governing melanoma cell behavior remain incompletely characterized. In the present study, we systematically profiled the inflammatory signaling pathway landscape across four independent melanoma cohorts and identified a panel of robust candidate prognostic genes through multicohort univariate Cox regression. We subsequently constructed and validated an optimal machine learning–based prognostic model that demonstrated superior predictive performance over existing published signatures, and we comprehensively characterized the immune microenvironment, genomic alteration landscape, and TMB interactions associated with risk stratification. Furthermore, CCNE1 was identified as the top oncogenic risk factor, and functional experiments demonstrated that CCNE1 knockdown suppressed melanoma cell migration and enhanced cell adhesion, collectively implicating CCNE1 as a promising therapeutic target in melanoma.

## 2. Methods

### 2.1. Data Collection and Cohort Curation

Four independent melanoma gene expression datasets were included in this study: three Gene Expression Omnibus (GEO) datasets (GSE19234 [18], GSE22153 [19], and GSE65904 [20]) and The Cancer Genome Atlas (TCGA) melanoma cohort (TCGA‐SKCM). For GEO datasets, raw expression matrices and corresponding clinical annotation files were downloaded from the NCBI GEO database (https://www.ncbi.nlm.nih.gov/geo/). For the TCGA cohort, RNA‐seq count data and clinical information were retrieved via the TCGAbiolinks R package. All continuous gene expression data were log_2_‐transformed where necessary. Samples lacking survival time or event status information were excluded from subsequent survival analyses.

### 2.2. Cross‐Cohort Data Harmonization and Batch Correction

To mitigate intercohort batch effects arising from differences in profiling platforms and sample processing, batch correction was performed using the ComBat algorithm implemented in the sva R package [21]. Principal component analysis (PCA) was subsequently applied to the integrated expression matrix to assess the extent of cross‐cohort sample mixing and validate the effectiveness of batch correction. The first two principal components (Dim1 and Dim2) were visualized, with samples colored by cohort of origin.

### 2.3. Inflammatory Pathway ssGSEA Scoring

A curated collection of 15 inflammatory signaling pathway gene sets was assembled based on established immunological databases and prior literature, encompassing core JAK‐STAT signaling, chemokine signaling, pathogen‐induced inflammation, general cytokine network, IL‐10 family signaling, metabolic inflammation, Toll‐like receptor signaling, IL‐6/STAT3 signaling, NF‐*κ*B signaling, IL‐2 family signaling, TGF‐*β* signaling, cell division in inflammation, IFN signaling, IL‐12/IL‐23 signaling, RTK variant–associated inflammation, and TNF signaling. ssGSEA was performed using the gene set variation analysis (GSVA) R package [22] (method = “ssgsea”) to quantify the activity of each pathway in individual samples. Mean ssGSEA scores across all samples within each cohort were calculated and visualized as bar plots with standard error of the mean (SEM) error bars.

### 2.4. Univariate Cox Proportional Hazards Regression Analysis

All genes constituting the 15 inflammatory signaling pathways were subjected to univariate Cox proportional hazards regression analysis in each of the four cohorts independently, using overall survival (OS) as the endpoint. The survival and survminer R packages were employed for this purpose. A gene was designated as a candidate prognostic gene only if it achieved statistical significance (*p* < 0.05) in all four cohorts simultaneously. The direction of the hazard ratio (HR) was required to be consistent across cohorts. Genes with HR > 1 were classified as hazardous factors, and those with HR < 1 were classified as protective factors. Results were visualized as a forest plot displaying the HR estimates and 95% confidence intervals for each candidate gene across the four cohorts.

### 2.5. Functional Enrichment Analysis

KEGG pathway enrichment analysis and Gene Ontology (GO) enrichment analysis were performed on the candidate prognostic gene set using the clusterProfiler R package. GO terms were categorized into three ontologies: biological process (BP), cellular component (CC), and molecular function (MF). Enrichment significance was assessed using a hypergeometric test, with a false discovery rate (FDR) threshold of < 0.05 applied for multiple testing correction. The top enriched terms from each category were visualized as horizontal bar plots with gene counts on the *x*‐axis.

### 2.6. Copy Number Variation (CNV) Analysis

Somatic copy number alteration (SCNA) data for the TCGA‐SKCM cohort were obtained from the UCSC Xena browser (https://xenabrowser.net/) as GISTIC2‐processed segment‐level copy number calls. For each candidate prognostic gene, the frequencies of copy number amplification (GISTIC2 threshold score ≥ 2) and deletion (GISTIC2 threshold score ≤ −2) events were calculated across all tumor samples. CNV frequency profiles were visualized as bar plots grouped by chromosomal location, with amplification frequencies displayed as positive values (orange) and deletion frequencies as negative values (blue).

### 2.7. Multialgorithm Machine Learning Framework

To identify the optimal prognostic signature from the candidate genes, a comprehensive machine learning framework was established by integrating 10 survival prediction algorithms: Lasso regression, Ridge regression, Elastic Net (Enet) (alpha = 0.1–0.9), Lasso‐Cox, stepwise Cox regression (forward, backward, and bidirectional selection), RSF, CoxBoost, GBM, Partial Least Squares Cox (plsCox), SuperPC, and Survival Support Vector Machine (Survival SVM). All possible pairwise combinations of these algorithms were enumerated, generating a large number of unique two‐step algorithm combinations. For each combination, the first algorithm was applied for feature selection or dimensionality reduction, and the second algorithm was used to construct the final risk score model. All models were trained in the TCGA cohort and evaluated in all four independent cohorts (TCGA, GSE19234, GSE22153, and GSE65904) using the concordance index (*C*‐index) as the primary performance metric. The algorithm combination achieving the highest mean *C*‐index across all four cohorts was designated as the final prognostic model. All machine learning analyses were performed using the mlr3, glmnet, randomForestSRC, CoxBoost, gbm, superpc, and survivalsvm R packages.

### 2.8. Benchmarking Against Published Prognostic Signatures

To comprehensively evaluate the competitive performance of our model relative to existing literature, we conducted a systematic literature search of PubMed for melanoma prognostic signature studies. For each identified publication, the originally reported prognostic gene set and corresponding risk score calculation formula were extracted. Risk scores were recalculated for each patient in all four cohorts (TCGA, GSE19234, GSE22153, and GSE65904) using the reported gene weights or coefficients. The *C*‐index of each published model was then computed in each cohort using the concordance function from the Hmisc R package, and models were ranked in descending order of the *C*‐index within each cohort. The performance of MY MODEL was simultaneously evaluated under identical conditions and positioned within the same ranking framework. Results were visualized as dot plots with models ranked along the *y*‐axis and *C*‐index values on the *x*‐axis.

### 2.9. Kaplan–Meier Survival Analysis and Risk Group Stratification

To evaluate the prognostic stratification capacity of the risk model, patients in each cohort were divided into high‐ and low‐risk groups using the median risk score of the TCGA training cohort as the cutoff threshold. OS differences between risk groups were assessed using Kaplan–Meier survival analysis, and statistical significance was evaluated by the two‐sided log‐rank test. Survival curves were generated using the survfit function from the survival R package and visualized using the survminer R package. The number at risk at each time point was annotated below the survival curves. A *p* value of < 0.05 was considered statistically significant.

### 2.10. PCA

To assess the transcriptomic discriminative power of the model gene expression signature, PCA was performed based on the normalized expression matrix of the model genes across all four cohorts (TCGA, GSE19234, GSE22153, and GSE65904). Prior to PCA, gene expression data were standardized using *z*‐score normalization. PCA was conducted using the prcomp function in R with scaling enabled. The first two principal components (PC1 and PC2) were used for two‐dimensional visualization, with the percentage of variance explained by each component annotated on the respective axes. Each patient was represented as a dot colored by risk group assignment (red: high risk; green: low risk), and gene contribution vectors (loadings) were overlaid as arrows in the biplot. All PCA plots were generated using the ggbiplot and ggplot2 R packages.

### 2.11. Immune Cell Infiltration Analysis

To characterize the immune cell infiltration landscape in the TCGA cohort, immune infiltration abundance estimates for a panel of immune cell types were retrieved from the TIMER database (https://timer.cistrome.org/) [23]. The retrieved immune infiltration data encompassed multiple immune cell populations estimated by TIMER. Differences in immune cell infiltration levels between high‐ and low‐risk groups were visualized as a heatmap using the pheatmap R package, with patients stratified by risk group and immune cell types arranged along the row axis. Color intensity reflects the relative immune infiltration abundance, scaled across patients.

### 2.12. ESTIMATE Analysis and Correlation With the Risk Score

To investigate the relationship between the risk score and the overall tumor microenvironment composition, stromal, immune, and combined microenvironment scores were estimated for all TCGA patients using the ESTIMATE algorithm, implemented via the estimate R package [24]. The ESTIMATE algorithm generates four scores for each tumor sample: StromalScore (reflecting stromal cell infiltration), ImmuneScore (reflecting immune cell infiltration), ESTIMATEScore (the sum of StromalScore and ImmuneScore, reflecting overall nontumor cell infiltration), and TumorPurity (estimated as a transformation of ESTIMATEScore, reflecting the proportion of tumor cells). Spearman correlation analysis was performed between the continuous risk score and each of the four ESTIMATE‐derived scores using the cor.test function in R. Scatter plots were generated with patients colored by risk group (red: high risk; green: low risk), and a linear regression fit with a 95% confidence interval was overlaid on each plot. The Spearman correlation coefficient (*r*) and Benjamini–Hochberg (BH)–adjusted *p* value (*q* value) were annotated on each panel. All visualizations were produced using the ggplot2 R package.

### 2.13. ssGSEA Scoring and Correlation Analysis

The activity scores of the cancer immunity cycle steps [25] and KEGG tumor‐related functional pathways were quantified for each TCGA patient using ssGSEA (method = “ssgsea”; kcdf = “Gaussian”; abs.ranking = TRUE), implemented via the GSVA R package. Spearman correlation analysis was performed between the continuous risk score and each activity score, and results were visualized as a correlation heatmap using the ggplot2 R package.

### 2.14. Immune‐Related Gene Expression Analysis

The expression levels of immune‐related genes spanning five functional categories (MHC Class I, MHC Class II, other MHC molecules, coinhibitors, and costimulators) were compared between the high‐ and low‐risk groups using the Wilcoxon rank‐sum test and visualized as violin plots using the ggpubr R package.

### 2.15. Somatic Mutation Landscape Analysis

Somatic mutation data in MAF format were downloaded from the TCGA data portal and analyzed using the maftools R package. Patients were stratified by the median risk score, and an OncoPrint waterfall plot was generated to visualize the top frequently mutated genes and copy number alterations (CNAs) in each risk group.

### 2.16. TMB Calculation and Combined Prognostic Stratification

TMB was calculated as the number of somatic nonsynonymous mutations per megabase using maftools. Patients were classified into high‐ and low‐TMB groups using the median TMB as the cutoff, then combined with risk group assignment to form four subgroups. OS differences were assessed by Kaplan–Meier analysis and the log‐rank test and visualized using the survminer R package.

### 2.17. CCNE1 Protein Expression Validation

Immunohistochemical staining images of CCNE1 in melanoma tumor tissue and normal skin tissue were retrieved from the Human Protein Atlas (HPA) database (https://www.proteinatlas.org/).

### 2.18. Prognostic Analysis of CCNE1 in Multiple Cohorts

Patients in the TCGA, GSE19234, GSE22153, and GSE65904 cohorts were stratified into high‐ and low‐CCNE1 expression groups using the median expression as the cutoff. OS differences were assessed by Kaplan–Meier analysis and the log‐rank test and visualized using the survminer R package.

### 2.19. Single‐Cell RNA Sequencing (scRNA‐seq) Analysis

Ten melanoma tumor samples from the GSE277165 scRNA‐seq dataset were ranked by the average CCNE1 expression level and divided into High_CCNE1 (top 5) and Low_CCNE1 (bottom 5) groups. Cell type annotation and tSNE dimensionality reduction were performed using the Seurat R package. Cell type proportions between the two groups were compared using the Wilcoxon rank‐sum test. Cell–cell communication analysis was conducted using the CellChat R package, and the number of inferred interactions and interaction strength were compared between groups.

### 2.20. Cell Functional Assays

Human melanoma cell lines A375 (RRID: CVCL_0132; *Homo sapiens*, male, skin melanoma) and SK‐MEL‐28 (RRID: CVCL_0526; *H. sapiens*, male, skin melanoma) were obtained from ATCC (American Type Culture Collection) and cultured in DMEM supplemented with 10% FBS and 1% penicillin/streptomycin at 37°C with 5% CO_2_. Both cell lines were authenticated by short tandem repeat (STR) profiling prior to use, showing > 95% match with reference profiles, and were confirmed to be free of mycoplasma contamination. CCNE1 was silenced by lentiviral transduction of short hairpin RNA (shRNA) targeting CCNE1 (sh‐CCNE1), with a nontargeting shRNA as the negative control (NC). Knockdown efficiency was validated by qRT‐PCR using the 2^−*ΔΔ*Ct^ method with GAPDH as the reference gene. For wound healing assays, a scratch was made in confluent cell monolayers, and the migration rate was calculated as the percentage of wound closure at 24 h. Cell migration was further assessed by Transwell assays (8‐*μ*m pore size, without Matrigel), in which migrated cells were fixed, stained with crystal violet, and counted at 10× magnification after 24 h. For cell adhesion assays, cells were seeded onto fibronectin‐coated plates and incubated for 30 min; adherent cells were then fixed, stained, and quantified. All experiments were performed in triplicate, and data are presented as mean ± SD. Statistical comparisons between two groups were performed using unpaired Student′s *t*‐test, with *p* < 0.05 considered statistically significant.

### 2.21. Mutation Subtype Classification and Immune Microenvironment Analysis

Somatic mutation MAF files were downloaded from the GDC data portal for the TCGA‐SKCM cohort using the TCGAbiolinks package and parsed with maftools. Samples were classified into four mutually exclusive subtypes in order of priority (BRAF > NRAS > NF1 > Triple‐WT) based on driver gene mutation status. Raw RNA‐seq data were TPM‐normalized, and the ESTIMATE algorithm was applied to calculate StromalScore, ImmuneScore, and ESTIMATEScore; overall group differences were assessed by the Kruskal–Wallis test and pairwise comparisons by the Wilcoxon rank‐sum test. GSVA was performed on log_2_(TPM + 1)‐normalized matrices using the GSVA package (maxDiff = TRUE) to score nine immune‐related pathways, including T cell exhaustion, cytotoxic T cells, regulatory T cells (Tregs), M1/M2 macrophage polarization, NK cell activity, immunosuppression, IFN‐*γ* response, and antigen presentation. Immune cell infiltration was quantified using the IOBR package, integrating four deconvolution algorithms—TIMER, CIBERSORT (100 permutations), xCell, and EPIC—with group differences in eight representative cell subsets evaluated by the Kruskal–Wallis test. Differential expression analysis was conducted using the limma linear modeling framework (lmFit + eBayes) with log_2_(TPM + 1) as input, comparing each of the three mutation subtypes against Triple‐WT; differentially expressed genes were defined by |log2*F* *C*| > 1.5 and BH‐adjusted *p* < 0.05.

### 2.22. CCNE1 Virtual Knockdown

Melanoma tumor cell subclusters were extracted using Seurat, and transcriptomic signature scores for four mutation subtypes—BRAF, NRAS, NF1, and Triple‐WT—were computed using the AddModuleScore function based on published subtype‐specific gene sets. Each cell was assigned to the subtype with the highest module score, and low‐confidence cells were filtered using a confidence threshold of *Δ*score ≥ 0.05 (difference between the highest and second‐highest scores). Annotated cells were then stratified by subtype and randomly downsampled (up to 800 cells per subtype). Among cells with nonzero CCNE1 expression, cells in the top 25th percentile were designated as the high‐expression group and those in the bottom 25th percentile as the low‐expression group to simulate a virtual knockdown. Differential expression of antigen presentation genes (HLA‐A/B/C, B2M, TAP1/2, and TAPBP) and immune checkpoint molecules (CD274, PDCD1LG2, PDCD1, TIGIT, and LAG3) between the two groups was assessed using the Wilcoxon rank‐sum test implemented in Seurat FindMarkers, performed both globally and stratified by mutation subtype. Multiple testing correction was applied using the BH method. All visualizations were generated using ggplot2.

## 3. Results

### 3.1. Inflammatory Pathway Landscape Reveals Immune‐Related Prognostic Signatures and Genomic Alterations

**Figure 1:**
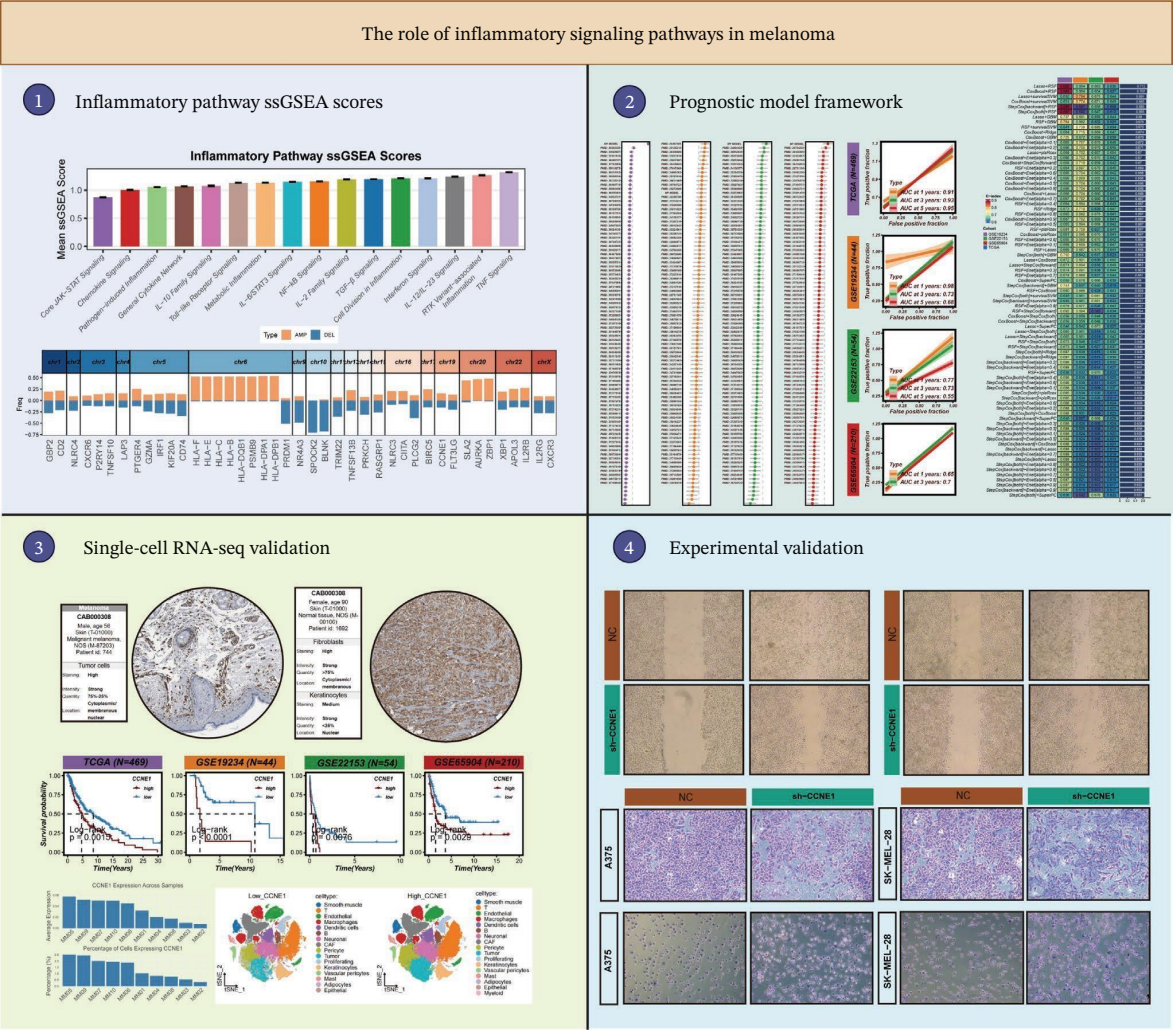
Overview of the study framework.

**Figure 2:**
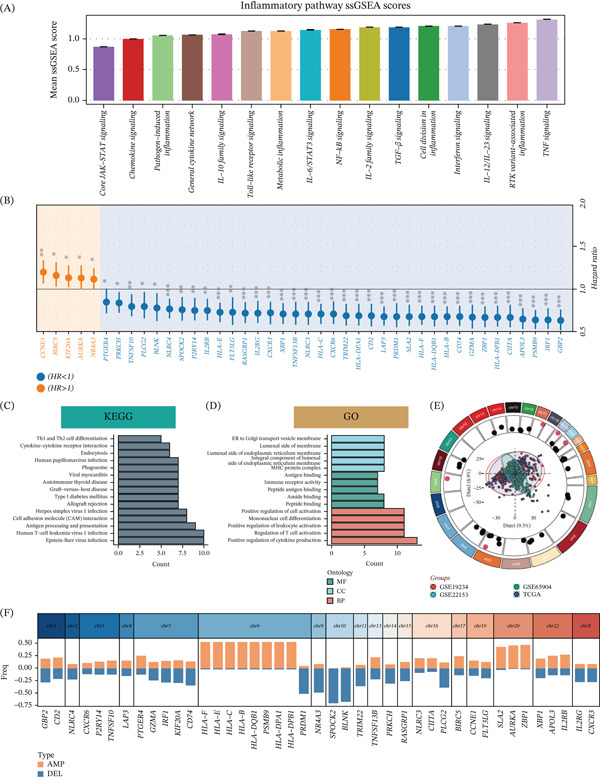
Inflammatory pathway activity, prognostic gene identification, functional annotation, chromosomal distribution, and copy number variation (CNV) landscape in melanoma. (A) Bar plot depicting the mean ssGSEA scores of 15 inflammatory signaling pathways across four independent melanoma cohorts (GSE19234, GSE22153, GSE65904, and TCGA). Error bars represent the standard error of the mean (SEM). Pathways are ordered along the *x*‐axis, and the *y*‐axis represents the mean ssGSEA enrichment score. (B) Forest plot displaying the hazard ratios (HRs) of candidate prognostic genes derived from the 15 inflammatory pathways, as determined by univariate Cox proportional hazards regression analysis performed simultaneously across all four cohorts. Orange dots indicate hazardous factors (HR > 1), and blue dots indicate protective factors (HR < 1). Dots represent the estimated HR, and horizontal lines represent 95% confidence intervals. Asterisks denote statistical significance ( ^∗^ *p* < 0.05;  ^∗∗^ *p* < 0.01;  ^∗∗∗^ *p* < 0.001). (C) Bar plot showing KEGG pathway enrichment analysis of the candidate prognostic genes. The *x*‐axis represents the gene count enriched in each pathway, and pathways are ranked by count. (D) Bar plot showing Gene Ontology (GO) enrichment analysis of the candidate prognostic genes, categorized by ontology: molecular function (MF) (green), cellular component (CC) (teal), and biological process (BP) (salmon). The *x*‐axis represents the gene count enriched in each GO term. (E) Circular chromosome plot illustrating the genomic localization of candidate prognostic genes across chromosomes. The embedded PCA scatter plot shows the distribution of samples from the four cohorts (GSE19234, GSE22153, GSE65904, and TCGA) along the first two principal components (Dim1: 9.5%; Dim2: 8.9%), demonstrating effective cross‐cohort data harmonization. (F) Bar plot depicting the CNV frequency of candidate prognostic genes across chromosomes. Orange bars represent amplifications (AMP), and blue bars represent deletions (DEL). The *y*‐axis indicates the frequency of CNV events, and genes are grouped and labeled by their respective chromosomal location on the *x*‐axis.

Figure 1 is the schematic overview of the research workflow of this study. To systematically characterize the inflammatory microenvironment in melanoma, we performed ssGSEA to score 15 curated inflammatory signaling pathways across four independent cohorts (GSE19234, GSE22153, GSE65904, and TCGA). As shown in Figure 2A, the majority of inflammatory pathways exhibited relatively high mean ssGSEA scores, with TNF signaling, IFN signaling, and IL‐12/IL‐23 signaling showing comparatively higher enrichment levels, whereas core JAK‐STAT signaling displayed relatively lower activity. These findings indicate that inflammatory signaling is broadly activated in melanoma, with notable heterogeneity in activity levels across distinct pathway types. To identify inflammatory pathway–related genes with robust prognostic significance, we extracted all component genes from the 15 pathways and simultaneously performed univariate Cox proportional hazards regression analysis across all four cohorts. Only genes reaching statistical significance (*p* < 0.05) in all four datasets were retained as candidate prognostic genes. As illustrated in the forest plot in Figure 2B, the candidate genes fell into two distinct prognostic categories: hazardous factors (HR > 1, orange), including CCNE1, BIRC5, KIF20A, AURKA, and NR4A3, whose high expression was associated with poorer survival outcomes; and protective factors (HR < 1, blue), comprising a larger set of immune‐related genes such as GBP2, IRF1, PSMB9, CIITA, and multiple HLA family members, whose high expression correlated with more favorable survival. The consistent prognostic directionality observed across all four independent cohorts underscores the robustness of these candidate genes as prognostic indicators. To gain functional insight into the biological roles of these prognostic candidates, we performed KEGG pathway and GO enrichment analyses (Figure 2C,D). KEGG analysis revealed significant enrichment in multiple immune‐related pathways, including Th1 and Th2 cell differentiation, cytokine–cytokine receptor interaction, and antigen processing and presentation, collectively highlighting the central importance of antigen presentation and adaptive immune regulation within the functional network defined by these genes. GO enrichment analysis yielded results highly concordant with the KEGG findings: at the CC level, candidate genes were significantly enriched in the MHC protein complex; at the MF level, enrichment was observed for antigen binding and immune receptor activity; and at the BP level, enrichment was detected for positive regulation of cytokine production, T cell activation, leukocyte activation, and mononuclear cell differentiation. These results collectively corroborate the integral role of these candidate genes in orchestrating antitumor immune responses. To characterize the chromosomal distribution of the candidate genes, we generated a circular chromosome localization plot (Figure 2E). The embedded PCA revealed that, despite the distinct origins of the four cohorts, samples exhibited substantial overlap along the primary axes of variation (Dim1: 9.5%; Dim2: 8.9%), indicating effective harmonization across datasets and validating the reliability of the cross‐cohort analytical framework. Finally, to explore the potential genomic mechanisms underlying the dysregulation of these prognostic genes, we analyzed their CNV profiles across chromosomes (Figure 2F). The analysis uncovered distinct patterns of SCNAs: genes located on Chromosome 6, including multiple HLA loci, predominantly harbored copy number amplifications. Notably, several hazardous factor genes—including BIRC5, CCNE1, and AURKA—exhibited recurrent amplification events, whereas a subset of immune‐protective genes were subject to copy number deletions, suggesting that CNV‐driven suppression of immune effector gene expression may represent a key mechanism of immune evasion in melanoma.

### 3.2. Development and Validation of an Optimal Machine Learning–Based Prognostic Model

**Figure 3:**
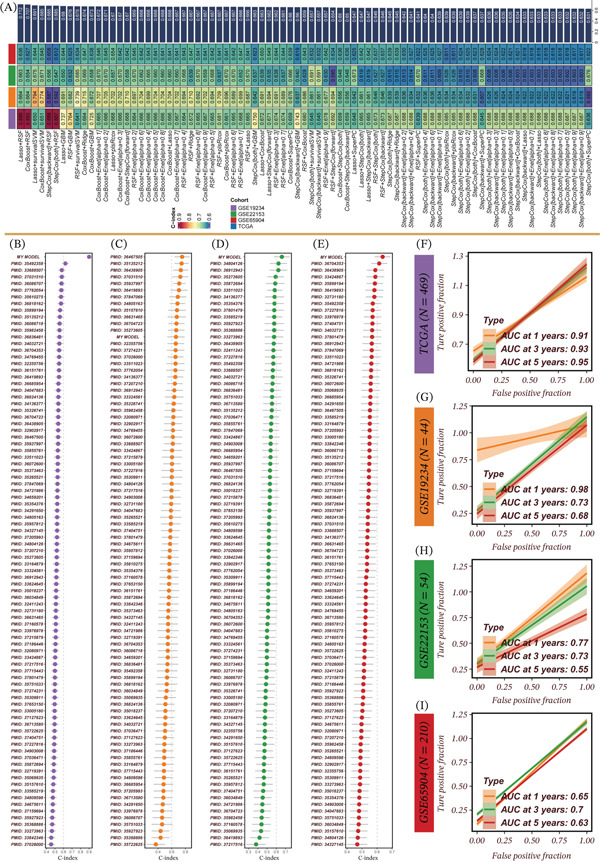
Construction of an optimal prognostic model via comprehensive machine learning algorithm integration and multicohort benchmarking against published signatures in melanoma. (A) Heatmap displaying the concordance index (*C*‐index) values of all algorithm combinations generated by pairwise permutation of 10 machine learning algorithms across four independent cohorts (TCGA, GSE19234, GSE22153, and GSE65904). Each row represents a unique algorithm combination, and each column represents a cohort. (B–E) Dot plots comparing the *C*‐index of MY MODEL against prognostic signatures from previously published melanoma studies retrieved from PubMed, evaluated in the TCGA (B), GSE19234 (C), GSE22153 (D), and GSE65904 (E) cohorts, respectively. (F–I) Time‐dependent receiver operating characteristic (ROC) curves evaluating the predictive accuracy of MY MODEL for 1‐, 3‐, and 5‐year overall survival in the TCGA (*N* = 469) (F), GSE19234 (*N* = 44) (G), GSE22153 (*N* = 54) (H), and GSE65904 (*N* = 210) (I) cohorts, respectively.

To construct an optimal prognostic signature from the candidate genes identified in the preceding analysis, we employed a comprehensive machine learning framework integrating 10 widely used survival prediction algorithms, generating a large number of unique algorithm combinations through pairwise permutation. These combinations encompassed Lasso regression, Ridge regression, Enet at multiple alpha values (0.1–0.9), Lasso‐Cox, stepwise Cox regression (forward, backward, and bidirectional), RSF, CoxBoost, GBM, plsCox, SuperPC, and Survival SVM, among others. Each algorithm combination was evaluated across all four independent cohorts (TCGA, GSE19234, GSE22153, and GSE65904) using the *C*‐index as the performance metric. As shown in the heatmap in Figure 3A, color intensity directly reflects the variation in predictive performance across algorithm combinations and cohorts. The combination yielding the highest mean *C*‐index across all four cohorts was selected as the final model (hereafter referred to as “MY MODEL”). This systematic and unbiased selection strategy ensures that the final model achieves optimal and robust predictive performance across heterogeneous datasets, effectively minimizing the risk of overfitting to any single cohort. To benchmark the predictive capacity of our model against existing published prognostic signatures, we systematically curated prognostic models from melanoma‐related studies indexed in PubMed, recalculated the *C*‐index of each published model across the same four cohorts using a unified analytical pipeline, and visualized the results as dot plots ranked by the *C*‐index value (Figure 3B–E). Notably, our model ranked first in three out of four cohorts—TCGA (Figure 3B), GSE22153 (Figure 3D), and GSE65904 (Figure 3E)—demonstrating a clear and consistent performance advantage over previously published signatures. In the GSE19234 cohort (Figure 3C), our model likewise achieved a highly competitive ranking. The sustained superiority of our model across multiple independent cohorts, particularly in larger datasets, provides compelling evidence for its robustness and generalizability relative to the existing literature. To further evaluate the time‐dependent discriminative accuracy of our prognostic model, we generated time‐dependent receiver operating characteristic (ROC) curves at 1‐, 3‐, and 5‐year time points for each of the four cohorts (Figure 3F–I). In the TCGA cohort (*N* = 469, Figure 3F), the model demonstrated excellent predictive performance, with area under the curve (AUC) values of 0.91, 0.93, and 0.95 at 1, 3, and 5 years, respectively, suggesting that predictive accuracy further improved with longer follow‐up horizons. In the GSE19234 cohort (*N* = 44, Figure 3G), AUC values were 0.98, 0.73, and 0.68 at 1, 3, and 5 years, respectively, with particularly outstanding short‐term prediction. In the GSE22153 cohort (*N* = 54, Figure 3H), AUC values of 0.77, 0.73, and 0.55 were achieved at 1, 3, and 5 years, respectively. In the GSE65904 cohort (*N* = 210, Figure 3I), AUC values were 0.65, 0.70, and 0.63 at 1, 3, and 5 years, respectively. Collectively, the consistently favorable AUC values across all four independent cohorts—particularly the outstanding performance in the TCGA and GSE19234 datasets—substantiate the strong and reliable prognostic discrimination capacity of our model, supporting its potential for clinical translation in melanoma survival prediction.

### 3.3. Prognostic Stratification and Immune Microenvironment Characterization

**Figure 4:**
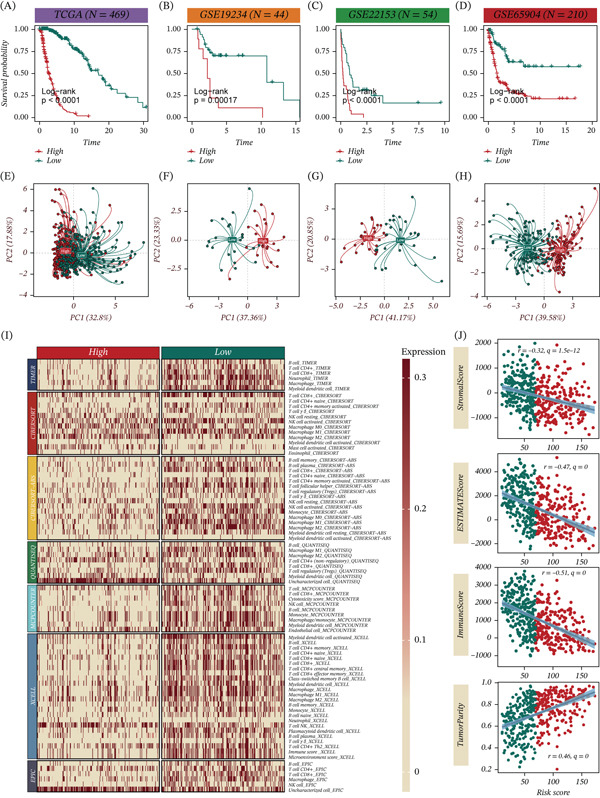
Prognostic stratification, transcriptomic discrimination, and tumor immune microenvironment characterization of the risk model across independent melanoma cohorts. (A–D) Kaplan–Meier survival curves comparing overall survival between high‐risk (red) and low‐risk (green) groups stratified by the median risk score in the TCGA (*N* = 469) (A), GSE19234 (*N* = 44) (B), GSE22153 (*N* = 54) (C), and GSE65904 (*N* = 210) (D) cohorts, respectively. Statistical significance was assessed by the log‐rank test; *p* values are annotated on each panel. (E–H) Principal component analysis (PCA) plots based on the expression profiles of model genes in the TCGA (E), GSE19234 (F), GSE22153 (G), and GSE65904 (H) cohorts, respectively. Each dot represents an individual patient, colored by risk group (red: high risk; green: low risk). The percentage of variance explained by PC1 and PC2 is indicated on the respective axes. Arrows represent the contribution vectors of individual model genes to the principal components. (I) Heatmap displaying the immune cell infiltration landscape in the TCGA cohort stratified by risk group (high risk: left; low risk: right), estimated using the TIMER database. Each row represents a specific immune cell type, and each column represents an individual patient. Color intensity reflects the relative abundance of immune infiltration, ranging from low (white) to high (dark red). (J) Scatter plots illustrating the Spearman correlations between the continuous risk score and ESTIMATE algorithm–derived scores in the TCGA cohort, including StromalScore (top), ESTIMATEScore (second), ImmuneScore (third), and TumorPurity (bottom). Each dot represents an individual patient, colored by risk group (red: high risk; green: low risk). The Spearman correlation coefficient (*r*) and adjusted *p* value (*q*) are annotated in each panel. The blue line with a shaded area represents the linear regression fit with a 95% confidence interval.

To evaluate the prognostic stratification capacity of MY MODEL, patients in each of the four cohorts were divided into high‐ and low‐risk groups based on the median risk score. Kaplan–Meier survival analysis with log‐rank testing revealed that high‐risk patients exhibited significantly worse OS compared with low‐risk patients across all four cohorts: TCGA (*N* = 469, log‐rank *p* < 0.0001, Figure 4A), GSE19234 (*N* = 44, log‐rank *p* = 0.00017, Figure 4B), GSE22153 (*N* = 54, log‐rank *p* < 0.0001, Figure 4C), and GSE65904 (*N* = 210, log‐rank *p* < 0.0001, Figure 4D). The consistent and highly significant survival differences observed across all four independent cohorts robustly validate the clinical prognostic value of MY MODEL, confirming its ability to effectively discriminate patients with distinct survival outcomes. To further assess the discriminative power of the model at the transcriptomic level, PCA was performed based on the expression profiles of the model genes in each cohort (Figure 4E–H). In all four cohorts, high‐ and low‐risk patients formed two clearly separated clusters in the two‐dimensional PCA space. The evident spatial segregation between risk groups in the PCA plots indicates that the model gene expression signature captures fundamental and biologically meaningful transcriptomic differences between high‐ and low‐risk patients, further corroborating the accuracy and biological relevance of the model. To characterize the immune microenvironment landscape associated with risk stratification, immune cell infiltration in the TCGA cohort was systematically assessed using quantitative immune infiltration estimates derived from the TIMER database (Figure 4I). Patients in the high‐risk group showed markedly lower levels of immune cell infiltration, indicating that elevated risk scores are accompanied by a suppressed TME. To further examine the relationship between the continuous risk score and the overall tumor microenvironment composition, Spearman correlation analyses were performed in the TCGA cohort between the risk score and ESTIMATE‐derived scores, including StromalScore, ESTIMATEScore, ImmuneScore, and TumorPurity (Figure 4J). The risk score was significantly negatively correlated with StromalScore (*r* = −0.32), ESTIMATEScore (*r* = −0.47), and ImmuneScore (*r* = −0.51) and positively correlated with TumorPurity (*r* = 0.46). Collectively, these results suggest that higher‐risk tumors tend to exhibit reduced stromal and immune components while displaying increased tumor cell purity, consistent with an immunosuppressive or “immune‐desert” microenvironment phenotype in the high‐risk group. This immune‐shielding pattern may contribute to immune evasion by limiting effective immune cell recruitment and compromising antitumor immune activity, thereby providing a plausible mechanistic basis for the worse prognosis observed in high‐risk patients. Moreover, such an immune infiltration landscape may have potential implications for immunotherapy response, as tumors with depleted immune activity are often less responsive to immune checkpoint blockade.

### 3.4. Immune Microenvironment Remodeling, Genomic Instability, and TMB‐Integrated Prognostic Stratification

**Figure 5:**
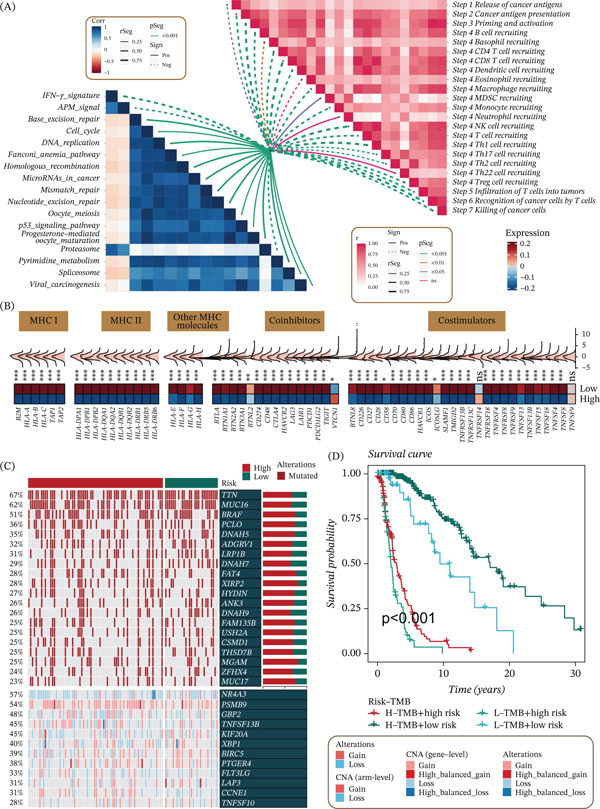
Association of the risk score with the cancer immunity cycle, immune‐related gene expression, somatic mutation landscape, and TMB‐based prognostic stratification in melanoma. (A) Correlation heatmap illustrating the Spearman correlations between the continuous risk score and ssGSEA‐derived activity scores of the cancer immunity cycle steps (right panel) and tumor‐related functional pathways (left panel) in the TCGA cohort. The cancer immunity cycle steps include Step 1 (release of cancer antigens) through Step 7 (killing of cancer cells). (B) Violin plots displaying the expression levels of immune‐related genes across five functional categories—MHC Class I, MHC Class II, other MHC molecules, coinhibitors, and costimulators—in the high‐risk (red) and low‐risk (green) groups of the TCGA cohort. Statistical significance between the two groups is indicated by asterisks above each gene ( ^∗^ *p* < 0.05;  ^∗∗^ *p* < 0.01;  ^∗∗∗^ *p* < 0.001). Abbreviation: ns, not significant. (C) OncoPrint mutation waterfall plot depicting the somatic alteration landscape of the most frequently mutated genes in the TCGA cohort, stratified by risk group (high risk: red bar; low risk: teal bar). Each column represents an individual patient, and each row represents a gene. The alteration types are color‐coded as follows: point mutations (Mutated, dark red), CNA arm‐level gain (Gain, pink), CNA arm‐level loss (Loss, light blue), CNA gene‐level high balanced gain (High_balanced_gain, red), and CNA gene‐level high balanced loss (High_balanced_loss, dark blue). The mutation frequency (%) for each gene is annotated on the left. (D) Kaplan–Meier survival curves comparing overall survival among four patient subgroups stratified by the combination of TMB level and risk score: H‐TMB+high risk (red), H‐TMB+low risk (dark green), L‐TMB+high risk (teal), and L‐TMB+low risk (light blue) in the TCGA cohort. Statistical significance was assessed by the log‐rank test (*p* < 0.001).

To further elucidate the relationship between the risk score and tumor immune function as well as related signaling pathways, ssGSEA was employed to quantify the activity scores of each step of the cancer immunity cycle and multiple tumor‐related functional pathways for each patient in the TCGA cohort. Spearman correlation analysis was then performed between these activity scores and the continuous risk score (Figure 5A). The risk score was significantly negatively correlated with all steps of the cancer immunity cycle, encompassing cancer antigen release, antigen presentation, T cell priming and activation, recruitment of multiple immune cell subsets, T cell infiltration into tumors, recognition of cancer cells by T cells, and cancer cell killing, indicating a systematic suppression of antitumor immune responses in high‐risk patients. Conversely, the risk score was significantly positively correlated with protumorigenic and DNA damage repair pathways, including cell cycle, DNA replication, Fanconi anemia pathway, homologous recombination, mismatch repair, nucleotide excision repair, proteasome, pyrimidine metabolism, and spliceosome, suggesting that high‐risk tumors are characterized by enhanced proliferative activity and heightened genomic instability. To further evaluate differences in immune‐related gene expression between risk groups, the expression levels of five categories of immune‐related genes—MHC Class I molecules, MHC Class II molecules, other MHC molecules, coinhibitory molecules, and costimulatory molecules—were systematically compared between the high‐ and low‐risk groups (Figure 5B). The results demonstrated that immune‐related genes were globally upregulated in the low‐risk group, particularly HLA family genes, immune checkpoint–related genes (including CD274, CTLA4, LAG3, TIGIT, HAVCR2, PDCD1, and PDCD1LG2), and multiple costimulatory molecules (including members of the TNFRSF and TNFSF families). These findings suggest that the low‐risk group harbors a more immunologically active tumor microenvironment, whereas the high‐risk group exhibits a phenotype of suppressed immune function. To characterize the genomic alteration landscape between risk groups, somatic mutation data from the TCGA cohort were used to generate an OncoPrint mutation waterfall plot (Figure 5C). Both risk groups exhibited predominantly point mutation–driven alteration patterns, accompanied by CNAs including arm‐level and gene‐level amplifications (Gain/High_balanced_gain) and deletions (Loss/High_balanced_loss). Notably, the high‐risk group demonstrated an overall higher mutation frequency, indicative of greater genomic instability. Given that TMB represents an important biomarker for predicting immunotherapy response, patients were further stratified by TMB level (high TMB vs. low TMB) and risk score (high risk vs. low risk) into four subgroups: H‐TMB+high risk, H‐TMB+low risk, L‐TMB+high risk, and L‐TMB+low risk. Kaplan–Meier survival analysis revealed significant differences in OS among the four subgroups (log‐rank *p* < 0.001, Figure 5D). The H‐TMB+low risk group demonstrated the most favorable prognosis, whereas the H‐TMB+high risk group exhibited the worst survival outcomes, suggesting that high mutational burden not only fails to confer survival benefit in the context of high‐risk scores but may further promote immune evasion or tumor progression. The L‐TMB+low risk group showed an intermediate‐to‐favorable prognosis, whereas L‐TMB+high risk patients also demonstrated poor survival. Collectively, these findings indicate that the combined stratification of risk score and TMB enables more refined prognostic subgrouping of melanoma patients, providing a more comprehensive framework for clinical prognostic assessment and immunotherapy decision‐making.

### 3.5. CCNE1 Is Overexpressed in Melanoma and Associated With Poor Prognosis and an Immunosuppressive Tumor Microenvironment

**Figure 6:**
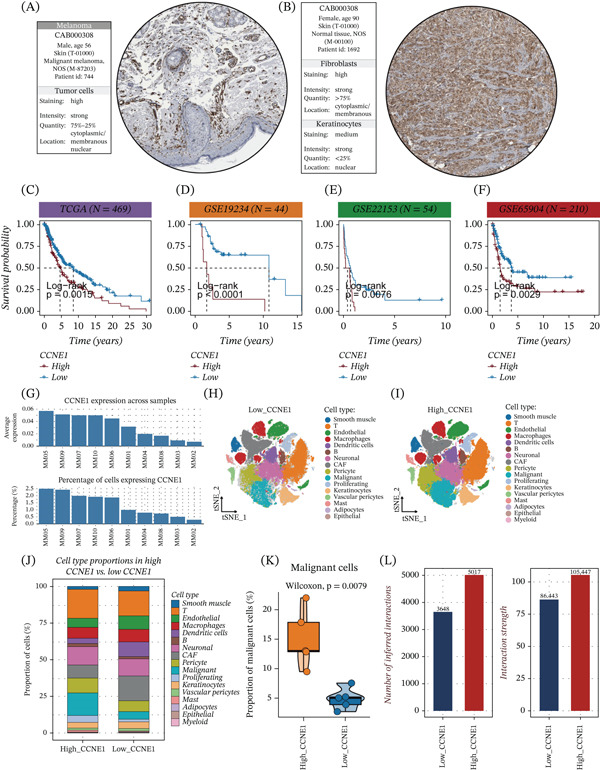
CCNE1 overexpression is associated with poor prognosis, tumor cell enrichment, and activated cell–cell communication in melanoma. (A, B) Representative IHC images of CCNE1 in melanoma tumor tissue (A) and normal skin tissue (B) from the Human Protein Atlas (HPA) database, showing higher CCNE1 protein expression in tumor cells. (C–F) Kaplan–Meier survival curves comparing overall survival between CCNE1 high‐ and low‐expression groups (stratified by median expression) in the TCGA (*N* = 469, *p* = 0.0015), GSE19234 (*N* = 44, *p* < 0.0001), GSE22153 (*N* = 54, *p* = 0.0076), and GSE65904 (*N* = 210, *p* = 0.0029) cohorts. (G) Bar plots showing the average CCNE1 expression level and the percentage of CCNE1‐expressing cells across 10 melanoma samples from the GSE277165 scRNA‐seq dataset. Samples ranked by CCNE1 expression were divided into High_CCNE1 (top 5) and Low_CCNE1 (bottom 5) groups. (H, I) tSNE plots displaying the cellular composition of the Low_CCNE1 (H) and High_CCNE1 (I) groups, with cells colored by annotated cell type. (J) Stacked bar plots comparing cell type proportions between the High_CCNE1 and Low_CCNE1 groups. (K) Violin plot comparing tumor cell proportions between the two groups (Wilcoxon test, *p* = 0.0079). (L) Bar plots showing the number of inferred cell–cell interactions and interaction strength in the Low_CCNE1 and High_CCNE1 groups as determined by CellChat analysis.

To further validate the association between model gene expression and the continuous risk score, Spearman correlation analysis was performed for all genes included in the prognostic model (Supporting Information 1: Figure S1). Among them, CCNE1 exhibited the strongest positive correlation with the risk score and had well‐established oncogenic functions, and it was therefore selected as the candidate gene for subsequent in‐depth investigation. To validate the protein expression pattern of CCNE1, immunohistochemical staining data were retrieved from the HPA database (Figure 6A,B). In melanoma tumor tissue (patient CAB000308, male, aged 56), CCNE1 exhibited strong positive staining with 25%–75% of tumor cells stained, localized to the cytoplasm/membrane and nucleus (Figure 6A). In normal skin tissue (patient CAB000308, female, aged 90), CCNE1 showed high expression in fibroblasts (strong intensity, > 75% of cells, cytoplasmic/membranous localization) and medium expression in keratinocytes (strong intensity, < 25% of cells, nuclear localization) (Figure 6B), collectively indicating prominent protein‐level overexpression of CCNE1 in melanoma tumor cells. To assess the prognostic significance of CCNE1 expression in melanoma, patients in four independent cohorts—TCGA (*N* = 469), GSE19234 (*N* = 44), GSE22153 (*N* = 54), and GSE65904 (*N* = 210)—were stratified into high‐ and low‐expression groups based on the median CCNE1 expression level, and Kaplan–Meier survival analysis was performed (Figure 6C–F). Across all four cohorts, patients with high CCNE1 expression demonstrated significantly shorter OS compared with those with low expression (TCGA: log‐rank *p* = 0.0015; GSE19234: log‐rank *p* < 0.0001; GSE22153: log‐rank *p* = 0.0076; GSE65904: log‐rank *p* = 0.0029), indicating that CCNE1 overexpression is a consistent and independent marker of poor prognosis in melanoma. To investigate the functional role of CCNE1 at the single‐cell level, 10 melanoma tumor samples from the GSE277165 scRNA‐seq dataset were selected and ranked by the average CCNE1 expression level, with the top 5 samples designated as the High_CCNE1 group and the bottom 5 as the Low_CCNE1 group (Figure 6G). tSNE dimensionality reduction visualization identified multiple cell types in both groups, including smooth muscle cells, T cells, endothelial cells, macrophages, dendritic cells, B cells, neuronal cells, CAFs, pericytes, tumor cells, proliferating cells, keratinocytes, vascular pericytes, mast cells, adipocytes, epithelial cells, and myeloid cells, with notable differences in overall cellular composition between the two groups (Figure 6H,I). Cell type proportion analysis revealed that the High_CCNE1 group harbored a significantly higher proportion of tumor cells, whereas the Low_CCNE1 group showed relatively higher proportions of immune cells, including dendritic cells (Figure 6J). Quantitative analysis of tumor cell proportions further confirmed a significantly greater tumor cell fraction in the High_CCNE1 group compared with the Low_CCNE1 group (Wilcoxon test, *p* = 0.0079), suggesting a close association between CCNE1 overexpression and tumor cell enrichment (Figure 6K). Cell–cell communication analysis demonstrated that both the number of inferred interactions (5017 vs. 3648) and interaction strength (105.447 vs. 86.443) were substantially higher in the High_CCNE1 group than in the Low_CCNE1 group, indicating a more active and complex intercellular communication network in CCNE1‐overexpressing tumor microenvironments (Figure 6L, Supporting Information 2: Figure S2).

### 3.6. CCNE1 Knockdown Inhibits Migration and Enhances Adhesion

**Figure 7:**
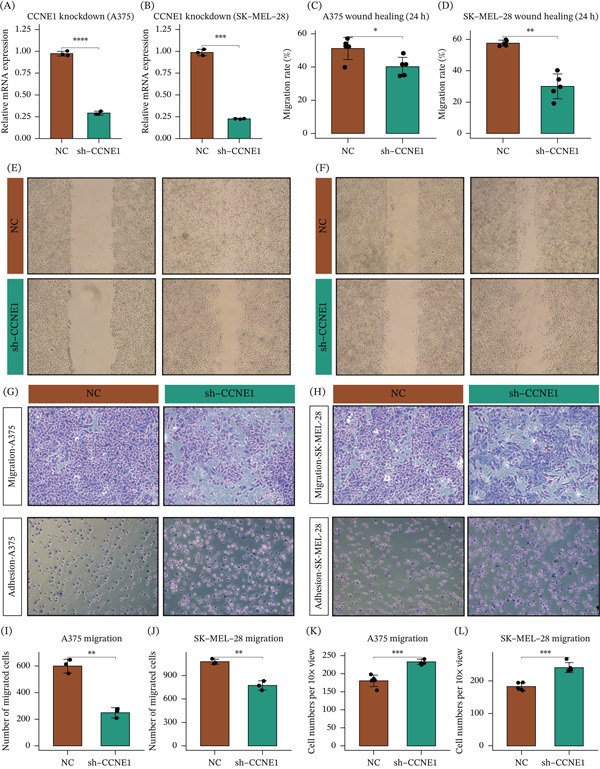
CCNE1 knockdown inhibits migration and enhances adhesion in melanoma cells. (A, B) qRT‐PCR showing CCNE1 mRNA expression in A375 (A) and SK‐MEL‐28 (B) cells after shRNA‐mediated knockdown (sh‐CCNE1) compared with negative control (NC) ( ^∗∗∗∗^ *p* < 0.0001;  ^∗∗∗^ *p* < 0.001). (C–F) Quantification (C, D) and representative images (E, F) of wound healing assays in A375 and SK‐MEL‐28 cells at 24 h ( ^∗^ *p* < 0.05;  ^∗∗^ *p* < 0.01). (G–J) Representative images (G, H) and quantification (I, J) of Transwell migration assays in A375 and SK‐MEL‐28 cells ( ^∗∗^ *p* < 0.01). (K, L) Quantification of cell adhesion assays in A375 (K) and SK‐MEL‐28 (L) cells ( ^∗∗∗^ *p* < 0.001). Data are presented as mean ± SD. Statistical significance was determined by unpaired Student′s *t*‐test.

To validate the functional role of CCNE1 in melanoma, we performed CCNE1 knockdown in two melanoma cell lines, A375 and SK‐MEL‐28, using shRNA. qRT‐PCR confirmed a significant reduction in CCNE1 mRNA expression in both sh‐CCNE1 groups compared with the NC (A375: *p* < 0.0001; SK‐MEL‐28: *p* < 0.001) (Figure 7A,B). Wound healing assays demonstrated that CCNE1 knockdown significantly reduced the migration rate of both A375 and SK‐MEL‐28 cells at 24 h compared with NC (A375: *p* < 0.05; SK‐MEL‐28: *p* < 0.01) (Figure 7C–F). Consistent with these findings, Transwell migration assays further confirmed that the number of migrated cells was markedly decreased in sh‐CCNE1 groups in both cell lines (A375: *p* < 0.01; SK‐MEL‐28: *p* < 0.01) (Figure 7G–J). In contrast, cell adhesion assays revealed that CCNE1 knockdown significantly increased cell adhesion in both A375 and SK‐MEL‐28 cells compared with NC (*p* < 0.001 for both) (Figure 7K,L). These results indicate that CCNE1 promotes melanoma cell migration while inhibiting cell adhesion, suggesting its oncogenic role in melanoma progression.

### 3.7. Distinct Immune Microenvironment Landscapes Across Four TCGA‐SKCM Mutation Subtypes

**Figure 8:**
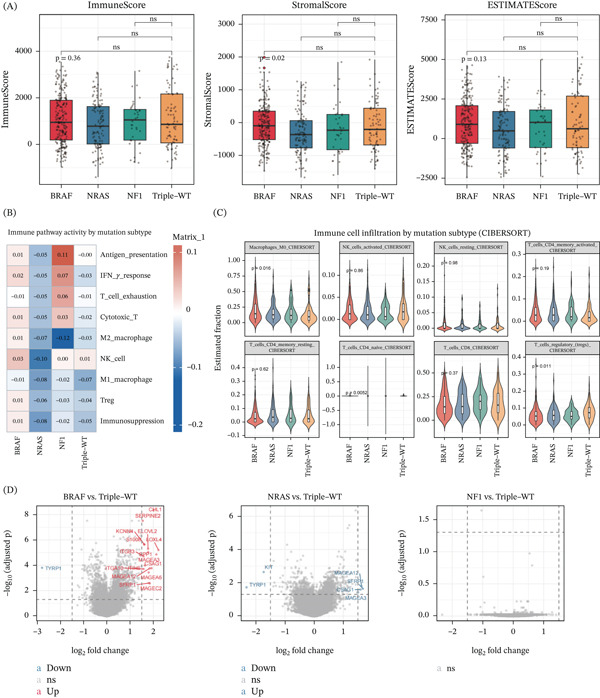
Multidimensional characterization of the immune microenvironment across four mutation subtypes in TCGA‐SKCM. (A) Comparison of ESTIMATE microenvironment scores—ImmuneScore, StromalScore, and ESTIMATEScore—across four mutation subtypes (BRAF, NRAS, NF1, and Triple‐WT). Boxplots overlaid with individual data points show the distribution of each sample; overall differences were assessed by the Kruskal–Wallis test and pairwise comparisons by the Wilcoxon rank‐sum test (ns: *p* ≥ 0.05). (B) Heatmap of GSVA enrichment scores for nine immune‐related pathways; values represent the mean score of all samples within each subtype; color gradient ranges from blue (negative enrichment) to red (positive enrichment) indicating pathway activity; rows are hierarchically clustered, and columns are arranged in fixed subtype order. (C) Violin plots of the estimated infiltration fractions of eight representative immune cell subsets derived from CIBERSORT deconvolution; embedded boxplots indicate the median and interquartile range; group differences were assessed by the Kruskal–Wallis test (*p* values shown within each panel). (D) Volcano plots of differentially expressed genes for each of the three mutation subtypes (BRAF, NRAS, and NF1) versus Triple‐WT; the *x*‐axis represents log_2_ fold change, and the *y*‐axis represents −log_10_(BH‐adjusted *p* value); red dots indicate significantly upregulated genes (log_2_FC > 1.5, adjusted *p* < 0.05), blue dots indicate significantly downregulated genes, and gray dots indicate nonsignificant genes; the top 20 most significant differentially expressed genes are labeled.

To systematically characterize the immune microenvironment across different driver mutation subtypes in the TCGA‐SKCM cohort, we performed a comprehensive analysis of BRAF‐, NRAS‐, and NF1‐mutant and Triple‐WT tumors across four dimensions: ESTIMATE scoring, immune pathway activity, cellular infiltration composition, and transcriptomic differences. ESTIMATE analysis revealed a statistically significant overall difference in StromalScore among the four subtypes (Kruskal–Wallis test,*p* = 0.02), with BRAF‐mutant tumors exhibiting the highest median StromalScore, whereas ImmuneScore and ESTIMATEScore did not reach significance (*p* = 0.36and*p* = 0.13, respectively), suggesting comparable overall immune infiltration levels but subtype‐specific differences in stromal composition (Figure 8A). GSVA pathway analysis demonstrated that NF1‐mutant tumors had the highest enrichment scores for antigen presentation (mean = 0.11) and IFN‐*γ* response (0.07), indicative of relatively stronger immune activation potential, whereas NRAS‐mutant tumors showed the lowest scores for NK cell activity (−0.10), reflecting a more pronounced immunosuppressive microenvironment; BRAF‐mutant tumors displayed near‐zero scores across most pathways, consistent with an immune‐neutral state (Figure 8B). CIBERSORT deconvolution identified significant subtype differences in M0 macrophages (*p* = 0.016) and Tregs (*p* = 0.011), with BRAF‐mutant tumors harboring the highest proportion of M0 macrophages, whereas effector immune cell subsets including CD8^+^ T cells and NK cells showed no significant differences across subtypes, suggesting relative conservation of effector immune function regardless of mutational background (Figure 8C). Transcriptomic analysis revealed that BRAF‐mutant tumors exhibited the greatest number of differentially expressed genes relative to Triple‐WT, with upregulated genes enriched in melanoma‐associated antigen families (MAGEA3, MAGEA6, and MAGEA12) and extracellular matrix remodeling genes (CHL1 and SERPINE2); TYRP1 was significantly downregulated in both BRAF‐ and NRAS‐mutant subtypes, potentially reflecting subtype‐specific dysregulation of the melanin synthesis pathway; NF1‐mutant tumors showed the fewest differentially expressed genes with limited effect sizes, suggesting that NF1 loss exerts a comparatively modest impact on global transcriptome remodeling relative to BRAF or NRAS mutation (Figure 8D).

### 3.8. CCNE1 Virtual Knockdown Upregulates Antigen Presentation and Suppresses Immune Checkpoint Expression in Tumor Cells

**Figure 9:**
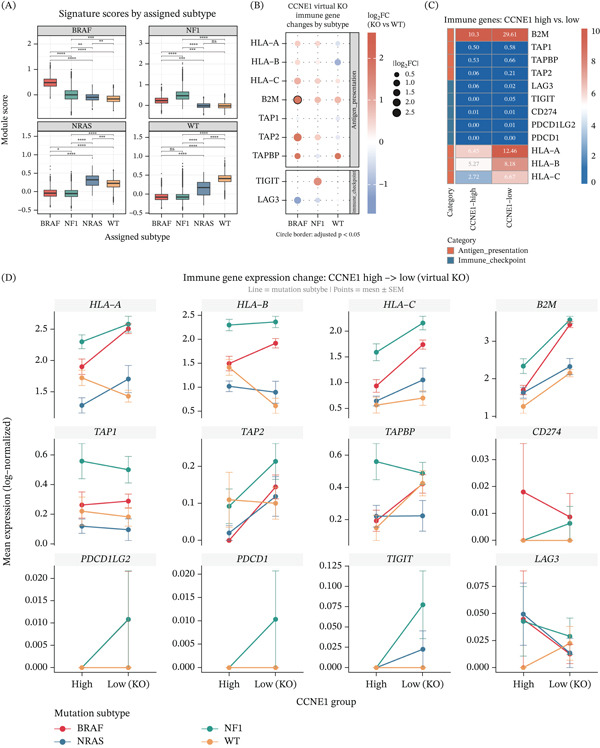
Impact of CCNE1 virtual knockdown on immune regulation in melanoma tumor cells. (A) Boxplots showing the distribution of mutation subtype signature scores across assigned subtype groups, validating the accuracy of subtype classification ( ^∗∗∗∗^ *p* < 0.0001;  ^∗∗∗^ *p* < 0.001;  ^∗∗^ *p* < 0.01;  ^∗^ *p* < 0.05). (B) Bubble plot showing differential expression of immune‐related genes across mutation subtypes under CCNE1 virtual knockdown (low vs. high expression). Bubble size indicates |log2*F* *C*|; color indicates the direction of fold change (blue: downregulated; red: upregulated). Black borders indicate statistically significant differences (adjusted *p* < 0.05, Benjamini–Hochberg correction). (C) Row‐normalized heatmap of mean immune gene expression between CCNE1‐high and CCNE1‐low groups. Left color bar denotes functional category (orange: antigen presentation; blue: immune checkpoint). (D) Faceted line plots showing subtype‐specific effects of CCNE1 virtual knockdown on immune gene expression. Data points represent group means ± SEM. Line colors indicate mutation subtypes (red: BRAF; blue: NRAS; green: NF1; orange: WT).

To investigate the role of CCNE1 in immune regulation within melanoma tumor cells, we constructed a CCNE1 virtual knockdown model using single‐cell transcriptomic data and systematically evaluated its effects on the antigen presentation pathway and immune checkpoint molecules. Mutation subtype assignment was performed by scoring cells against subtype‐specific gene signatures for BRAF, NRAS, NF1, and Triple‐WT subtypes. Boxplot validation confirmed that cells assigned to each subtype exhibited substantially higher corresponding signature scores relative to other subtypes (Figure 9A), supporting the reliability of the classification, with BRAF‐assigned cells showing a median BRAF signature score of approximately 0.5 and NF1‐assigned cells displaying a comparable NF1 score. Comparison of the top and bottom CCNE1 expression quartiles revealed subtype‐dependent immune gene alterations. Bubble plot analysis (Figure 9B) showed that in the BRAF mutation subtype, B2M (log_2_FC ≈ −2.1, adjusted *p* < 0.05) and TAP2 (log_2_FC ≈ −1.8, adjusted *p* < 0.05) were significantly upregulated in CCNE1‐high cells, indicating that CCNE1 knockdown enhances antigen presentation capacity in this subtype. Concurrently, LAG3 expression was significantly reduced upon CCNE1 knockdown in BRAF‐subtype cells (adjusted *p* < 0.05). In the NF1 subtype, TIGIT was significantly elevated in the CCNE1‐low group (adjusted *p* < 0.05), suggesting subtype‐specific heterogeneity in CCNE1‐mediated immune checkpoint regulation. At the global level, row‐normalized heatmap analysis (Figure 9C) revealed systematic differences in immune gene expression between the CCNE1‐high and CCNE1‐low groups: nearly all antigen presentation genes (HLA‐A/B/C, B2M, TAP1, TAP2, and TAPBP) and immune checkpoint molecules (CD274, PDCD1LG2, PDCD1, and TIGIT) exhibited higher relative expression in the CCNE1‐low group, whereas LAG3 was enriched in the CCNE1‐high group, suggesting that high CCNE1 expression may mediate immune suppression through LAG3 upregulation. Faceted line plot analysis further delineated the heterogeneity of virtual knockdown effects across mutation subtypes (Figure 9D). Among antigen presentation genes, NF1‐subtype cells showed the most pronounced upregulation of HLA‐A, HLA‐B, HLA‐C, and B2M following CCNE1 knockdown, indicating the greatest potential for enhanced immunogenicity in this subtype; BRAF‐subtype cells also demonstrated notable upregulation, whereas WT‐subtype cells exhibited comparatively modest changes. Regarding immune checkpoint molecules, PDCD1LG2, PDCD1, and TIGIT were markedly upregulated in NF1‐subtype cells with low CCNE1 expression, whereas minimal changes were observed in other subtypes, implying that the NF1 mutational context may confer specificity to CCNE1‐mediated immune checkpoint regulation. Collectively, these findings suggest that suppression of CCNE1 expression may exert a proimmunogenic effect by enhancing antigen presentation, with this effect being most prominent in the NF1 mutation subtype.

## 4. Discussion

In the present study, we systematically characterized the inflammatory signaling pathway landscape across four independent melanoma cohorts and constructed a robust machine learning–based prognostic model with superior predictive performance. Our findings demonstrate that inflammatory signaling is broadly activated in melanoma, with TNF signaling, IFN signaling, and IL‐12/IL‐23 signaling exhibiting the highest enrichment levels, consistent with prior reports implicating these pathways as central regulators of the melanoma immune microenvironment [26]. The identification of two functionally distinct categories of prognostic genes—hazardous factors (CCNE1, BIRC5, KIF20A, AURKA, and NR4A3) and immune‐protective factors (GBP2, IRF1, PSMB9, CIITA, and multiple HLA family members)—is particularly noteworthy. The hazardous genes are predominantly associated with cell cycle regulation, chromosomal segregation, and proliferative signaling, whereas the protective genes converge on antigen presentation, MHC complex assembly, and adaptive immune activation, a dichotomy that mirrors the fundamental tension between tumor cell–intrinsic proliferative drive and host immune surveillance [5, 27]. Notably, CNV analysis revealed that several hazardous factors, including BIRC5, CCNE1, and AURKA, undergo recurrent genomic amplification, whereas a subset of immune‐protective genes are subject to copy number deletions, suggesting that SCNAs represent a key genomic mechanism through which melanoma cells simultaneously enhance proliferative signaling and evade immune recognition [28, 29]. The consistent prognostic directionality of these candidate genes across all four independent cohorts, each derived from distinct patient populations and profiling platforms, provides compelling evidence for their biological robustness and potential clinical utility as prognostic indicators in melanoma.

The machine learning–based prognostic model constructed in the present study demonstrated consistently superior predictive performance across all four independent cohorts compared with previously published signatures, achieving AUC values as high as 0.95 in the TCGA cohort. This performance advantage likely reflects several methodological strengths. First, the use of an ensemble framework integrating 10 complementary survival prediction algorithms—including Lasso‐Cox, RSF, CoxBoost, and GBM—enables the model to capture both linear and nonlinear associations between gene expression features and survival outcomes, substantially reducing the risk of overfitting inherent to single‐algorithm approaches [30]. Second, the systematic multicohort selection strategy, which identifies the algorithm combination achieving the highest mean *C*‐index across all four cohorts, ensures that model selection is guided by cross‐dataset generalizability rather than single‐cohort optimization, a critical yet frequently overlooked consideration in prognostic model development. Kaplan–Meier survival analysis confirmed highly significant survival stratification in all four cohorts, and PCA visualization demonstrated clear transcriptomic separation between high‐ and low‐risk groups, collectively validating that the risk score captures biologically meaningful and clinically relevant molecular differences. The systematic benchmarking against published models using a unified analytical pipeline further substantiates the superiority of our model, mitigating potential biases introduced by inconsistent evaluation methodologies across studies. Together, these findings establish our machine learning framework as a robust and generalizable prognostic tool for melanoma, with clear potential for clinical translation.

A central finding of the present study is the comprehensive characterization of the immune microenvironment and genomic alteration landscape associated with risk stratification. High‐risk patients exhibited markedly suppressed immune cell infiltration, reduced cancer immunity cycle activity across all seven steps—from cancer antigen release to cancer cell killing—and significant downregulation of MHC Class I and II molecules, immune checkpoint molecules, and costimulatory molecules, collectively indicative of a profoundly immunosuppressive or immune‐desert tumor microenvironment phenotype [25, 31]. These observations are mechanistically consistent with the negative correlations observed between the risk score and ESTIMATE‐derived ImmuneScore and StromalScore, alongside its positive correlation with TumorPurity [24], suggesting that immune exclusion and stromal depletion are cardinal features of the high‐risk tumor microenvironment. Conversely, the high‐risk group was characterized by significant enrichment of DNA damage repair pathways—including homologous recombination, mismatch repair, Fanconi anemia pathway, and nucleotide excision repair—as well as enhanced cell cycle and DNA replication activity, indicating a state of elevated replication stress and genomic instability that likely drives the accumulation of somatic mutations and further shapes the immunosuppressive niche [32]. The integration of TMB with risk stratification revealed a nuanced relationship between mutational burden and prognosis: although high TMB is generally associated with increased neoantigen load and improved immunotherapy response [33], our data demonstrate that high‐TMB patients in the high‐risk group exhibited the worst OS, suggesting that the immunosuppressive microenvironment characteristic of high‐risk tumors may negate the potential immunogenic benefit of elevated mutational burden. This finding has important clinical implications, indicating that TMB alone is insufficient for prognostic assessment in melanoma and that its combinatorial integration with immune microenvironment–based risk scores provides a more refined and clinically actionable stratification framework, potentially informing patient selection for ICI therapy [34].

The identification of CCNE1 as the top‐ranked oncogenic risk gene in our prognostic model and the subsequent functional characterization of its role in melanoma constitute a major translational contribution of the present study. CCNE1 encodes Cyclin E1, a critical regulator of the G1/S cell cycle transition that forms complexes with CDK2 to phosphorylate RB and drive S phase entry [35]. Consistent with its known oncogenic functions, CCNE1 was found to be overexpressed in melanoma tumor tissue at the protein level, as validated by immunohistochemical data from the HPA database, and its high expression independently predicted significantly shorter OS across all four cohorts. These findings extend prior reports of CCNE1 amplification and overexpression in other solid tumors—including breast, ovarian, and gastric cancers—to the melanoma context, reinforcing its broad oncogenic relevance [36–38]. Beyond its canonical proliferative functions, our scRNA‐seq analysis revealed that CCNE1‐high tumors harbored a significantly greater proportion of tumor cells alongside reduced immune cell fractions, particularly dendritic cells, and exhibited substantially higher intercellular communication complexity, suggesting that CCNE1 overexpression is associated with a protumorigenic microenvironment characterized by immune exclusion and enhanced tumor–stroma crosstalk. The depletion of dendritic cells observed in CCNE1‐high tumors is particularly noteworthy, as dendritic cells serve as central orchestrators of antitumor immunity through antigen presentation and T cell priming. Tumor‐intrinsic cell cycle dysregulation driven by CCNE1 overexpression may impair dendritic cell recruitment and maturation via suppression of key chemokine axes such as CCL4–CCR5 and CXCL10–CXCR3, thereby attenuating cytotoxic T lymphocyte infiltration and fostering an immunosuppressive niche [39].

A particularly novel dimension of the present study is the systematic dissection of immune microenvironment heterogeneity across the four major melanoma mutation subtypes—BRAF, NRAS, NF1, and Triple‐WT—and the demonstration that the oncogenic and immunomodulatory effects of CCNE1 are contingent upon mutational context. Our analyses revealed that NF1‐mutant tumors exhibited the highest enrichment of antigen presentation pathways and IFN‐*γ* signaling, suggesting an inherently more immunogenic baseline that may confer greater sensitivity to immune checkpoint inhibition. In contrast, NRAS‐mutant tumors displayed the most pronounced immunosuppressive phenotype, characterized by minimal NK cell activity, whereas BRAF‐mutant tumors occupied an immune‐neutral state with elevated stromal infiltration and M0 macrophage accumulation. These subtype‐specific immune landscapes are consistent with the distinct downstream signaling consequences of each driver mutation: NF1 loss leads to broad RAS hyperactivation with pleiotropic effects on IFN response genes, whereas oncogenic NRAS and BRAF mutations preferentially channel signaling through MEK‐ERK to suppress immunostimulatory cytokine production. Critically, virtual knockdown modeling using scRNA‐seq data demonstrated that CCNE1 suppression exerted its strongest proimmunogenic effects—upregulation of HLA‐A/B/C, B2M, TAP1/2, and TAPBP, alongside immune checkpoint downregulation—in NF1‐mutant tumor cells, whereas LAG3 downregulation was most prominent in BRAF‐mutant cells. These findings suggest that the therapeutic benefit of CCNE1 targeting may be amplified in specific mutational contexts and that integrating mutation subtype information with CCNE1 expression status could help identify patient subpopulations most likely to benefit from CDK2 inhibitor–based combination immunotherapy strategies.

Functionally, shRNA‐mediated knockdown of CCNE1 in A375 and SK‐MEL‐28 melanoma cell lines significantly inhibited cell migration in both wound healing and Transwell assays while concomitantly enhancing cell adhesion, indicating that CCNE1 promotes a migratory and invasive phenotype while suppressing adhesion‐mediated tumor cell anchorage. These observations align with emerging evidence that cyclin–CDK complexes regulate cytoskeletal dynamics and focal adhesion turnover beyond their canonical nuclear functions [40], and they suggest that the promigratory role of CCNE1 in melanoma may involve remodeling of the actin cytoskeleton and modulation of integrin‐mediated adhesion signaling.

Collectively, these findings position CCNE1 as a multifunctional oncogenic driver in melanoma that promotes tumor progression through coordinate regulation of cell cycle advancement, immune microenvironment remodeling, and enhanced cell motility, underscoring its potential as both a prognostic biomarker and a therapeutic target. From a clinical translational perspective, the therapeutic targeting of CCNE1 has gained considerable momentum in recent years, primarily through the development of selective CDK2 inhibitors. Several CDK2 inhibitors, including INX‐315 and PF‐07104091, have entered early‐phase clinical trials across multiple tumor types characterized by CCNE1 amplification or overexpression, demonstrating preliminary evidence of antitumor activity and manageable safety profiles [41]. Future studies employing in vivo models and pharmacological CDK2 inhibition will be essential to fully delineate the therapeutic window and mechanistic basis of CCNE1 targeting in melanoma.

## Author Contributions

Chong Mao and Guobin Chen contributed equally to this work and share first authorship.

## Funding

No funding was received for this manuscript.

## Ethics Statement

This study did not involve the collection of human tissue samples, animal experiments, or any direct interaction with human participants. All data analyzed in this study were obtained exclusively from publicly available databases, including GEO, TCGA, HPA, and TIMER. Therefore, no ethical approval was required for this study.

## Conflicts of Interest

The authors declare no conflicts of interest.

## Supporting Information

Additional supporting information can be found online in the Supporting Information section.

## Supporting information


**Supporting Information 1** Figure S1: Spearman correlation between model gene expression and the continuous risk score.

**Supporting Information 2** Figure S2: Cell–cell communication networks in the low‐CCNE1 and high‐CCNE1 groups.

## Data Availability

The bulk RNA sequencing datasets analyzed in this study are publicly available in the Gene Expression Omnibus (GEO) repository (https://www.ncbi.nlm.nih.gov/geo/) under accession numbers GSE19234, GSE22153, and GSE65904 and in The Cancer Genome Atlas (TCGA) database (https://portal.gdc.cancer.gov/). The single‐cell RNA sequencing dataset used for CCNE1 functional characterization is publicly accessible in GEO under accession number GSE277165. Immunohistochemical protein expression data for CCNE1 were retrieved from the Human Protein Atlas (HPA) database (https://www.proteinatlas.org/). Immune cell infiltration data were obtained from the TIMER database (https://timer.cistrome.org/). All original code and analytical pipelines used in this study are available from the corresponding authors upon reasonable request.

## References

[1] Reschke R. , Budde P. , Zucht H. D. , Mangana J. , Dummer R. , Pfoehler C. , Wistuba-Hamprecht K. , Weide B. , Hakim-Meibodi L. E. , Meier F. , Schulz C. , Richter J. , Bräutigam M. , Gutjahr C. , Schulz-Knappe P. , and Hassel J. C. , Autoantibodies as Predictors for Immune-Related Adverse Events in Checkpoint Inhibition Therapy of Metastatic Melanoma, Journal for ImmunoTherapy of Cancer. (2026) 14, no. 1, e013814, 10.1136/jitc-2025-013814. 41592893 PMC12853468

[2] Arnold M. , Singh D. , Laversanne M. , Vignat J. , Vaccarella S. , Meheus F. , Cust A. E. , de Vries E. , Whiteman D. C. , and Bray F. , Global Burden of Cutaneous Melanoma in 2020 and Projections to 2040, JAMA Dermatology. (2022) 158, no. 5, 495–503, 10.1001/jamadermatol.2022.0160, 35353115. 35353115 PMC8968696

[3] Robert C. , Schachter J. , Long G. V. , Arance A. , Grob J. J. , Mortier L. , Daud A. , Carlino M. S. , McNeil C. , Lotem M. , Larkin J. , Lorigan P. , Neyns B. , Blank C. U. , Hamid O. , Mateus C. , Shapira-Frommer R. , Kosh M. , Zhou H. , Ibrahim N. , Ebbinghaus S. , and Ribas A. , Pembrolizumab Versus Ipilimumab in Advanced Melanoma, New England Journal of Medicine. (2015) 372, no. 26, 2521–2532, 10.1056/NEJMoa1503093. 25891173

[4] Gaughan E. M. , Kim M. , Mendez I. , Rao A. D. , Wei M. , So A. , Zhong X. , Berking C. , Jiang R. , Kim T. M. , Dalle S. , Robert C. , Danson S. , Alam S. , Charles J. , Davies T. , Debus D. , Dzienis M. , Frazer R. , Gebhardt C. , Geidel G. , Hassel J. C. , Hansen I. , Heppt M. V. , Hildebrandt L. , Isaacs J. M. , Suh K. J. , Keam B. , Kim Y. J. , Lesimple T. , Saiag P. , Delibes A. , Barnett R. , Krepler C. , Gandhi K. , Qizilbash N. , Shui I. M. , Tan X. L. , and Sullivan R. J. , Resistance to Anti-PD-1 Immunotherapy for Stage III and IV Melanoma: A Global Chart Review Study, Journal for ImmunoTherapy of Cancer. (2026) 14, no. 3, e014564, 10.1136/jitc-2025-014564, 41786455. 41786455 PMC12970133

[5] Hanahan D. and Weinberg R. A. , Hallmarks of Cancer: The Next Generation, Cell. (2011) 144, no. 5, 646–674, 10.1016/j.cell.2011.02.013. 21376230

[6] Grivennikov S. I. , Greten F. R. , and Karin M. , Immunity, Inflammation, and Cancer, Cell. (2010) 140, no. 6, 883–899, 10.1016/j.cell.2010.01.025, 20303878. 20303878 PMC2866629

[7] Pansky A. , Hildebrand P. , Fasler-Kan E. , Baselgia L. , Ketterer S. , Beglinger C. , and Heim M. H. , Defective Jak-STAT Signal Transduction Pathway in Melanoma Cells Resistant to Growth Inhibition by Interferon-Alpha, International Journal of Cancer. (2000) 85, no. 5, 720–725, 10.1002/(SICI)1097-0215(20000301)85:5<720::AID-IJC20>3.0.CO;2-O, 10699955. 10699955

[8] Gong Z. , Du M. , Li Y. , Ye B. , Huang Y. , Gong H. , Wang W. , Chen L. , Ding Z. , and Zhang P. , Machine Learning Identifies TIME Subtypes Linking EGFR Mutations and Immune States in Lung Adenocarcinoma, NPJ Digital Medicine. (2025) 8, no. 1, 10.1038/s41746-025-02172-2, 41299024. PMC12749019 41299024

[9] Zhang P. , Liang X. , Ye B. , Wang X. , Wang Y. , Gong Z. , Huang Y. , Liu J. , Huang C. , and Luo P. , Metabolic Reprogramming Signature Predicts Immunotherapy Efficacy in Lung Adenocarcinoma: Targeting SLC25A1 to Overcome Immune Resistance, Chinese Journal of Cancer Research. (2025) 37, no. 6, 1000–1019, 10.21147/j.issn.1000-9604.2025.06.11, 41536497. 41536497 PMC12796613

[10] Zhang P. , Wu X. , Wang D. , Zhang M. , Zhang B. , and Zhang Z. , Unraveling the Role of Low-Density Lipoprotein-Related Genes in Lung Adenocarcinoma: Insights Into Tumor Microenvironment and Clinical Prognosis, Environmental Toxicology. (2024) 39, no. 10, 4479–4495, 10.1002/tox.24230, 38488684. 38488684

[11] Barbie D. A. , Tamayo P. , Boehm J. S. , Kim S. Y. , Moody S. E. , Dunn I. F. , Schinzel A. C. , Sandy P. , Meylan E. , Scholl C. , Fröhling S. , Chan E. M. , Sos M. L. , Michel K. , Mermel C. , Silver S. J. , Weir B. A. , Reiling J. H. , Sheng Q. , Gupta P. B. , Wadlow R. C. , Le H. , Hoersch S. , Wittner B. S. , Ramaswamy S. , Livingston D. M. , Sabatini D. M. , Meyerson M. , Thomas R. K. , Lander E. S. , Mesirov J. P. , Root D. E. , Gilliland D. G. , Jacks T. , and Hahn W. C. , Systematic RNA Interference Reveals That Oncogenic KRAS-Driven Cancers Require TBK1, Nature. (2009) 462, no. 7269, 108–112, 10.1038/nature08460, 19847166. 19847166 PMC2783335

[12] Zhang P. , Feng J. , Rui M. , Xie J. , Zhang L. , and Zhang Z. , Integrating Machine Learning and Single-Cell Analysis to Uncover Lung Adenocarcinoma Progression and Prognostic Biomarkers, Journal of Cellular and Molecular Medicine. (2024) 28, no. 13, e18516, 10.1111/jcmm.18516, 38958577. 38958577 PMC11221317

[13] Zhang P. , Yang Z. , Liu Z. , Zhang G. , Zhang L. , Zhang Z. , and Fan J. , Deciphering Lung Adenocarcinoma Evolution: Integrative Single-Cell Genomics Identifies the Prognostic Lung Progression Associated Signature, Journal of Cellular and Molecular Medicine. (2024) 28, no. 11, e18408, 10.1111/jcmm.18408, 38837585. 38837585 PMC11149493

[14] Samstein R. M. , Lee C. H. , Shoushtari A. N. , Hellmann M. D. , Shen R. , Janjigian Y. Y. , Barron D. A. , Zehir A. , Jordan E. J. , Omuro A. , Kaley T. J. , Kendall S. M. , Motzer R. J. , Hakimi A. A. , Voss M. H. , Russo P. , Rosenberg J. , Iyer G. , Bochner B. H. , Bajorin D. F. , Al-Ahmadie H. A. , Chaft J. E. , Rudin C. M. , Riely G. J. , Baxi S. , Ho A. L. , Wong R. J. , Pfister D. G. , Wolchok J. D. , Barker C. A. , Gutin P. H. , Brennan C. W. , Tabar V. , Mellinghoff I. K. , DeAngelis L. M. , Ariyan C. E. , Lee N. , Tap W. D. , Gounder M. M. , D′Angelo S. P. , Saltz L. , Stadler Z. K. , Scher H. I. , Baselga J. , Razavi P. , Klebanoff C. A. , Yaeger R. , Segal N. H. , Ku G. Y. , DeMatteo R. P. , Ladanyi M. , Rizvi N. A. , Berger M. F. , Riaz N. , Solit D. B. , Chan T. A. , and Morris L. G. T. , Tumor Mutational Load Predicts Survival After Immunotherapy Across Multiple Cancer Types, Nature Genetics. (2019) 51, no. 2, 202–206, 10.1038/s41588-018-0312-8, 30643254. 30643254 PMC6365097

[15] Gu Y. , Sun M. , Fang H. , Shao F. , Lin C. , Liu H. , Li H. , He H. , Li R. , Wang J. , Zhang H. , and Xu J. , Impact of Clonal TP53 Mutations With Loss of Heterozygosity on Adjuvant Chemotherapy and Immunotherapy in Gastric Cancer, British Journal of Cancer. (2024) 131, no. 8, 1320–1327, 10.1038/s41416-024-02825-1, 39217196. 39217196 PMC11473753

[16] Tang X. Y. , Zhang R. Z. , Feng Z. B. , Zhou Y. L. , Du W. G. , Shu C. , Shen Y. , Li M. C. , Cai J. C. , Yan X. L. , Ma N. , and Zhao J. B. , FGL1-Mediated Lymph Node Metastasis in Stage T1 Non-Small Cell Lung Cancer: Therapeutic Targeting, Experimental Hematology & Oncology. (2025) 14, no. 1, 10.1186/s40164-025-00709-5, 41024301. PMC12481761 41024301

[17] Hydbring P. , Malumbres M. , and Sicinski P. , Non-Canonical Functions of Cell Cycle Cyclins and Cyclin-Dependent Kinases, Nature Reviews Molecular Cell Biology. (2016) 17, no. 5, 280–292, 10.1038/nrm.2016.27, 27033256. 27033256 PMC4841706

[18] Bogunovic D. , O′Neill D. W. , Belitskaya-Levy I. , Vacic V. , Yu Y. L. , Adams S. , Darvishian F. , Berman R. , Shapiro R. , Pavlick A. C. , Lonardi S. , Zavadil J. , Osman I. , and Bhardwaj N. , Immune Profile and Mitotic Index of Metastatic Melanoma Lesions Enhance Clinical Staging in Predicting Patient Survival, Proceedings of the National Academy of Sciences of the United States of America. (2009) 106, no. 48, 20429–20434, 10.1073/pnas.0905139106, 19915147. 19915147 PMC2787158

[19] Jönsson G. , Busch C. , Knappskog S. , Geisler J. , Miletic H. , Ringnér M. , Lillehaug J. R. , Borg A. , and Lønning P. E. , Gene Expression Profiling-Based Identification of Molecular Subtypes in Stage IV Melanomas With Different Clinical Outcome, Clinical Cancer Research. (2010) 16, no. 13, 3356–3367, 10.1158/1078-0432.CCR-09-2509, 20460471. 20460471

[20] Cabrita R. , Lauss M. , Sanna A. , Donia M. , Skaarup Larsen M. , Mitra S. , Johansson I. , Phung B. , Harbst K. , Vallon-Christersson J. , van Schoiack A. , Lövgren K. , Warren S. , Jirström K. , Olsson H. , Pietras K. , Ingvar C. , Isaksson K. , Schadendorf D. , Schmidt H. , Bastholt L. , Carneiro A. , Wargo J. A. , Svane I. M. , and Jönsson G. , Tertiary Lymphoid Structures Improve Immunotherapy and Survival in Melanoma, Nature. (2020) 577, no. 7791, 561–565, 10.1038/s41586-019-1914-8, 31942071. 31942071

[21] Leek J. T. , Johnson W. E. , Parker H. S. , Jaffe A. E. , and Storey J. D. , The sva Package for Removing Batch Effects and Other Unwanted Variation in High-Throughput Experiments, Bioinformatics. (2012) 28, no. 6, 882–883, 10.1093/bioinformatics/bts034, 22257669. 22257669 PMC3307112

[22] Hänzelmann S. , Castelo R. , and Guinney J. , GSVA: Gene Set Variation Analysis for Microarray and RNA-seq Data, BMC Bioinformatics. (2013) 14, no. 1, 10.1186/1471-2105-14-7, 23323831. PMC3618321 23323831

[23] Li T. , Fu J. , Zeng Z. , Cohen D. , Li J. , Chen Q. , Li B. , and Liu X. S. , TIMER2.0 for Analysis of Tumor-Infiltrating Immune Cells, Nucleic Acids Research. (2020) 48, no. W1, W509–w514, 10.1093/nar/gkaa407, 32442275. 32442275 PMC7319575

[24] Yoshihara K. , Shahmoradgoli M. , Martínez E. , Vegesna R. , Kim H. , Torres-Garcia W. , Treviño V. , Shen H. , Laird P. W. , Levine D. A. , Carter S. L. , Getz G. , Stemke-Hale K. , Mills G. B. , and Verhaak R. G. , Inferring Tumour Purity and Stromal and Immune Cell Admixture From Expression Data, Nature Communications. (2013) 4, no. 1, 10.1038/ncomms3612, 24113773. PMC3826632 24113773

[25] Chen D. S. and Mellman I. , Oncology Meets Immunology: The Cancer-Immunity Cycle, Immunity. (2013) 39, no. 1, 1–10, 10.1016/j.immuni.2013.07.012, 23890059. 23890059

[26] Bald T. , Quast T. , Landsberg J. , Rogava M. , Glodde N. , Lopez-Ramos D. , Kohlmeyer J. , Riesenberg S. , van den Boorn-Konijnenberg D. , Hömig-Hölzel C. , Reuten R. , Schadow B. , Weighardt H. , Wenzel D. , Helfrich I. , Schadendorf D. , Bloch W. , Bianchi M. E. , Lugassy C. , Barnhill R. L. , Koch M. , Fleischmann B. K. , Förster I. , Kastenmüller W. , Kolanus W. , Hölzel M. , Gaffal E. , and Tüting T. , Ultraviolet-Radiation-Induced Inflammation Promotes Angiotropism and Metastasis in Melanoma, Nature. (2014) 507, no. 7490, 109–113, 10.1038/nature13111, 24572365. 24572365

[27] Dunn G. P. , Old L. J. , and Schreiber R. D. , The Three Es of Cancer Immunoediting, Annual Review of Immunology. (2004) 22, no. 1, 329–360, 10.1146/annurev.immunol.22.012703.104803, 15032581. 15032581

[28] Davoli T. , Xu A. W. , Mengwasser K. E. , Sack L. M. , Yoon J. C. , Park P. J. , and Elledge S. J. , Cumulative Haploinsufficiency and Triplosensitivity Drive Aneuploidy Patterns and Shape the Cancer Genome, Cell. (2013) 155, no. 4, 948–962, 10.1016/j.cell.2013.10.011, 24183448. 24183448 PMC3891052

[29] Rooney M. S. , Shukla S. A. , Wu C. J. , Getz G. , and Hacohen N. , Molecular and Genetic Properties of Tumors Associated With Local Immune Cytolytic Activity, Cell. (2015) 160, no. 1-2, 48–61, 10.1016/j.cell.2014.12.033, 25594174. 25594174 PMC4856474

[30] Binder H. and Schumacher M. , Allowing for Mandatory Covariates in Boosting Estimation of Sparse High-Dimensional Survival Models, BMC Bioinformatics. (2008) 9, no. 1, 10.1186/1471-2105-9-14, 18186927. PMC2245904 18186927

[31] Hegde P. S. and Chen D. S. , Top 10 Challenges in Cancer Immunotherapy, Immunity. (2020) 52, no. 1, 17–35, 10.1016/j.immuni.2019.12.011. 31940268

[32] Alexandrov L. B. , Nik-Zainal S. , Wedge D. C. , Aparicio S. A. , Behjati S. , Biankin A. V. , Bignell G. R. , Bolli N. , Borg A. , Børresen-Dale A. L. , Boyault S. , Burkhardt B. , Butler A. P. , Caldas C. , Davies H. R. , Desmedt C. , Eils R. , Eyfjörd J. E. , Foekens J. A. , Greaves M. , Hosoda F. , Hutter B. , Ilicic T. , Imbeaud S. , Imielinski M. , Jäger N. , Jones D. T. , Jones D. , Knappskog S. , Kool M. , Lakhani S. R. , López-Otín C. , Martin S. , Munshi N. C. , Nakamura H. , Northcott P. A. , Pajic M. , Papaemmanuil E. , Paradiso A. , Pearson J. V. , Puente X. S. , Raine K. , Ramakrishna M. , Richardson A. L. , Richter J. , Rosenstiel P. , Schlesner M. , Schumacher T. N. , Span P. N. , Teague J. W. , Totoki Y. , Tutt A. N. J. , Valdés-Mas R. , van Buuren M. M. , Veer L. v. ’t. , Vincent-Salomon A. , Waddell N. , Yates L. R. , Australian Pancreatic Cancer Genome Initiative, ICGC Breast Cancer Consortium, ICGC MMML-Seq Consortium, ICGC PedBrain , Zucman-Rossi J. , Futreal P. A. , McDermott U. , Lichter P. , Meyerson M. , Grimmond S. M. , Siebert R. , Campo E. , Shibata T. , Pfister S. M. , Campbell P. J. , and Stratton M. R. , Signatures of Mutational Processes in Human Cancer, Nature. (2013) 500, no. 7463, 415–421, 10.1038/nature12477, 23945592. 23945592 PMC3776390

[33] Yarchoan M. , Hopkins A. , and Jaffee E. M. , Tumor Mutational Burden and Response Rate to PD-1 Inhibition, New England Journal of Medicine. (2017) 377, no. 25, 2500–2501, 10.1056/NEJMc1713444, 29262275. 29262275 PMC6549688

[34] Cristescu R. , Mogg R. , Ayers M. , Albright A. , Murphy E. , Yearley J. , Sher X. , Liu X. Q. , Lu H. , Nebozhyn M. , Zhang C. , Lunceford J. K. , Joe A. , Cheng J. , Webber A. L. , Ibrahim N. , Plimack E. R. , Ott P. A. , Seiwert T. Y. , Ribas A. , McClanahan T. K. , Tomassini J. E. , Loboda A. , and Kaufman D. , Pan-Tumor Genomic Biomarkers for PD-1 Checkpoint Blockade-Based Immunotherapy, Science. (2018) 362, no. 6411, 10.1126/science.aar3593, 30309915. PMC6718162 30309915

[35] Hwang H. C. and Clurman B. E. , Cyclin E in Normal and Neoplastic Cell Cycles, Oncogene. (2005) 24, no. 17, 2776–2786, 10.1038/sj.onc.1208613. 15838514

[36] Dai S. , Li L. , Guo G. , Peng Y. , Yuan H. , and Li J. , CCNE1 Stabilizes ANLN by Counteracting FZR1-Mediated the Ubiquitination Modification to Promotes Triple Negative Breast Cancer Cell Stemness and Progression, Cell Death Discovery. (2025) 11, no. 1, 10.1038/s41420-025-02518-5, 40346052. PMC12064766 40346052

[37] Li H. , Sheng J. J. , Zheng S. A. , Liu P. W. , Wu N. , Zeng W. J. , Li Y. H. , and Wang J. , Platinum-Resistant Ovarian Cancer: From Mechanisms to Treatment Strategies, Genes & Diseases. (2026) 13, no. 2, 101801, 10.1016/j.gendis.2025.101801, 41376855. 41376855 PMC12688694

[38] Saito-Adachi M. , Hama N. , Totoki Y. , Nakamura H. , Arai Y. , Hosoda F. , Rokutan H. , Yachida S. , Kato M. , Fukagawa A. , and Shibata T. , Oncogenic Structural Aberration Landscape in Gastric Cancer Genomes, Nature Communications. (2023) 14, no. 1, 10.1038/s41467-023-39263-1, 37349325. PMC10287692 37349325

[39] Williford J. M. , Ishihara J. , Ishihara A. , Mansurov A. , Hosseinchi P. , Marchell T. M. , Potin L. , Swartz M. A. , and Hubbell J. A. , Recruitment of CD103(+) Dendritic Cells via Tumor-Targeted Chemokine Delivery Enhances Efficacy of Checkpoint Inhibitor Immunotherapy, Science Adventure. (2019) 5, no. 12, eaay1357, 10.1126/sciadv.aay1357, 31844672. PMC6905870 31844672

[40] Kapadia N. and Nurse P. , Spatiotemporal Orchestration of Mitosis by Cyclin-Dependent Kinase, Nature. (2025) 643, no. 8074, 1391–1399, 10.1038/s41586-025-09172-y, 40562936. 40562936 PMC12310531

[41] Dietrich C. , Trub A. , Ahn A. , Taylor M. , Ambani K. , Chan K. T. , Lu K. H. , Mahendra C. A. , Blyth C. , Coulson R. , Ramm S. , Watt A. C. , Matsa S. K. , Bisi J. , Strum J. , Roberts P. , and Goel S. , INX-315, a Selective CDK2 Inhibitor, Induces Cell Cycle Arrest and Senescence in Solid Tumors, Cancer Discovery. (2024) 14, no. 3, 446–467, 10.1158/2159-8290.CD-23-0954, 38047585. 38047585 PMC10905675

