# Supplementary figures and images for: Driver Mutation Subtypes Differentially Shape Immune Evasion Landscapes in Melanoma: An AI‐Driven Inflammatory Pathway Model Implicating CCNE1
Source: Hum Mutat. 2026 Jun 27;2026:6776070. doi: 10.1155/humu/6776070 (PMC13309799; doi:10.1155/humu/6776070)

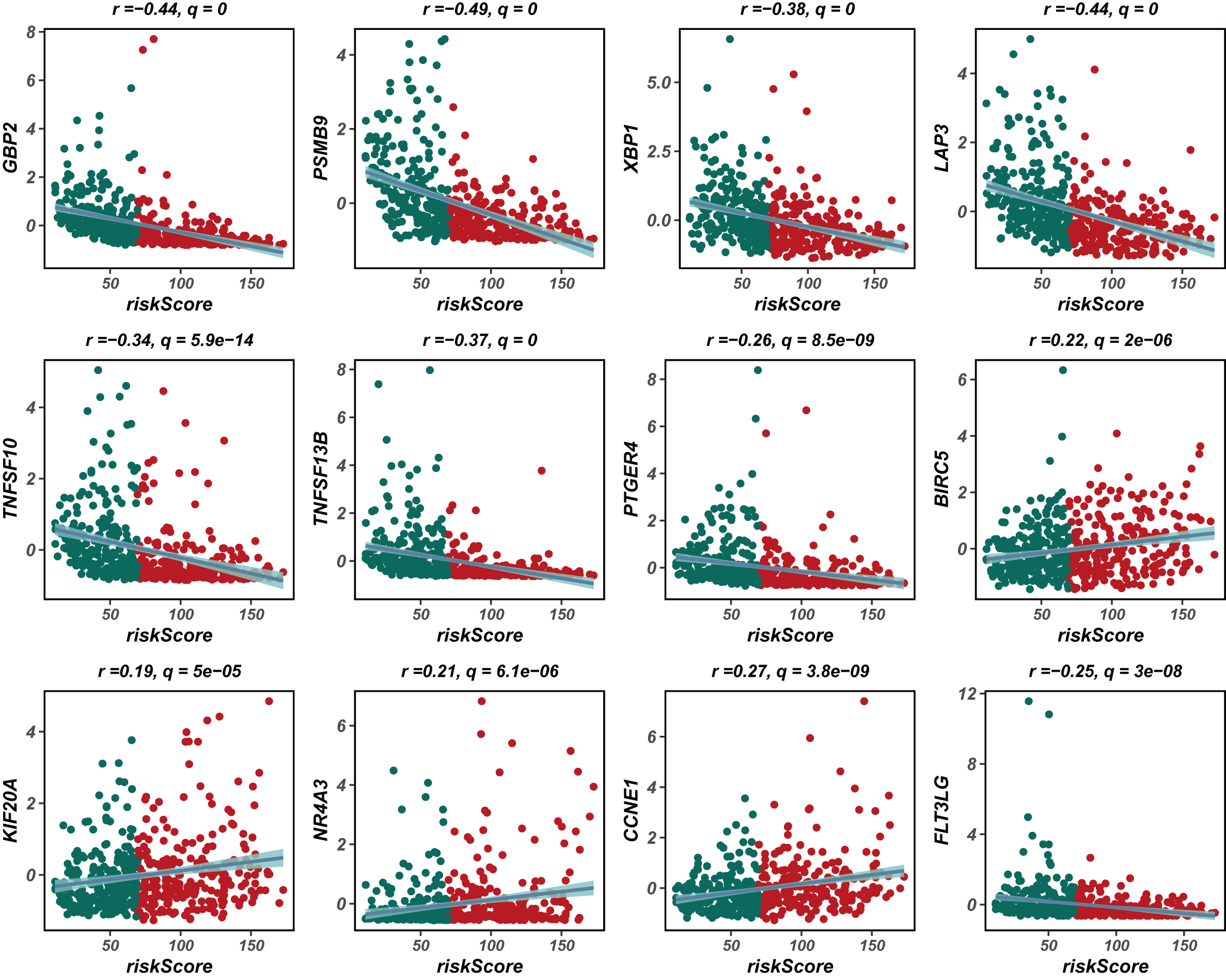

Supplement: Supplementary file 1 — Supporting Information 1 Figure S1: Spearman correlation between model gene expression and the continuous risk score. [file HUMU-2026-6776070-s001.tif]

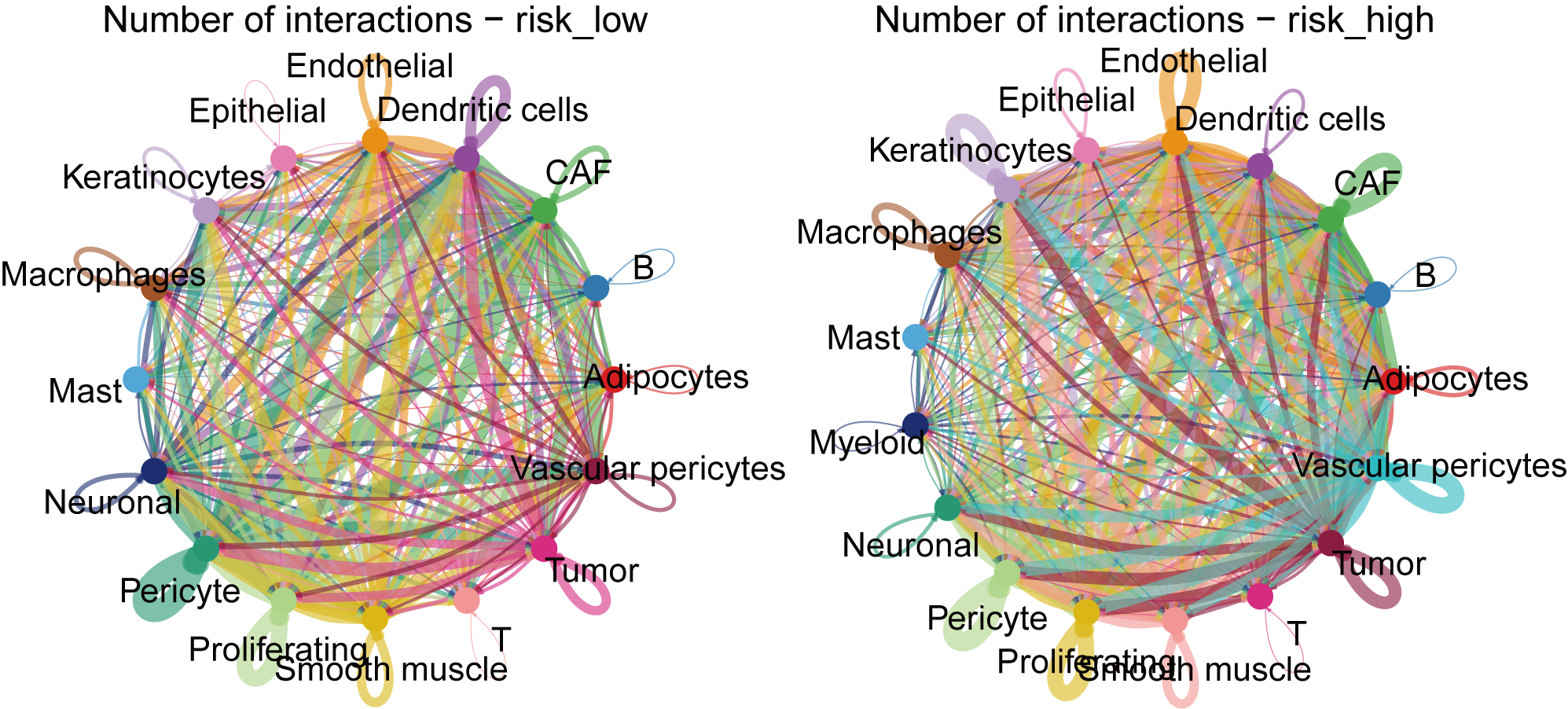

Supplement: Supplementary file 2 — Supporting Information 2 Figure S2: Cell–cell communication networks in the low‐CCNE1 and high‐CCNE1 groups. [file HUMU-2026-6776070-s002.tif]
